# Eicosanoid biosynthesis influences the virulence of *Candida parapsilosis*

**DOI:** 10.1080/21505594.2018.1475797

**Published:** 2018-07-27

**Authors:** Tanmoy Chakraborty, Ernst Thuer, Marieke Heijink, Renáta Tóth, László Bodai, Csaba Vágvölgyi, Martin Giera, Toni Gabaldón, Attila Gácser

**Affiliations:** a Department of Microbiology, University of Szeged, Szeged, Hungary; b Centre for Genomic Regulation (CRG), Barcelona Institute of Science and Technology, Barcelona, Spain; c Department of Experimental and Health Sciences, Universitat Pompeu Fabra (UPF), Barcelona, Catalonia, Spain; d Center for Proteomics and Metabolomics, Leiden University Medical Center, Leiden, The Netherlands; e Department of Biochemistry and Molecular Biology, University of Szeged, Szeged, Hungary; f Institució Catalana de Recerca i Estudis Avançats (ICREA), Barcelona, Spain

**Keywords:** *Candida parapsilosis*, fungal eicosanoids, immunomodulation, host-pathogen interaction, virulence

## Abstract

Lipid mediators, derived from arachidonic acid metabolism, play an important role in immune regulation. The functions of bioactive eicosanoids range from modulating cytokine signaling and inflammasome formation to anti-inflammatory and pro-resolving activities. Human pathogenic fungi such as *Candida albicans, Candida parapsilosis, Cryptococcus neoformans* and *Aspergillus fumigatus* have been shown to produce such lipid mediators, associated with their virulence. To date, investigations into the molecular mechanisms of fungal eicosanoid biosynthesis in different species have revealed that several genes are associated with prostaglandin production. However, these routes remain uncharacterized in *C. parapsilosis* with early results suggesting it uses pathways distinct from those found in *C. albicans*. Therefore, we aimed to identify and characterize *C. parapsilosis* genes involved in eicosanoid biosynthesis. Following arachidonic acid treatment of *C. parapsilosis* cells, we identified several genes interfering with prostaglandin production. Out of the identified genes, homologues of a multi copper oxidase (*FET3*), an Acyl-CoA thiolase (*POT1*) and an Acyl-CoA oxidase (*POX1-3*) were found to play a significant role in prostaglandin synthesis. Furthermore, all three genes were confirmed to enhance *C. parapsilosis* pathogenicity, as the corresponding deletion mutants were cleared more efficiently by human macrophages and induced higher levels of pro-inflammatory cytokines. In addition, the mutants were less virulent than the wild-type strain in a mouse model of systemic infection. Taken together, we identified three genes that regulate eicosanoid biosynthesis in *C. parapsilosis* and impact the fungus’ virulence.

## Introduction

Eicosanoids, are bioactive lipid molecules derived from the 20-carbon poly-unsaturated fatty acid, arachidonic acid. Prostaglandins and leukotrienes are two major groups of eicosanoids produced in almost all mammalian cells. Their production is initiated by phospholipases that mediate the release of arachidonic acid from the cell membrane and their activity is generally facilitated by binding to G-protein coupled receptors (GPCRs) and peroxisomal proliferator-activated receptors (PPARs) in different cells. Lipid mediators play a major role in inflammasome activation and the maintenance of cellular homoeostasis and in some cases, they act as pro-resolving/anti-inflammatory immune modulators (e.g. lipoxins). Three major enzymatic pathways are known to regulate eicosanoid synthesis, namely cyclooxygenase (*COX*), lipoxygenase (*LOX*) and cytochrome P450 proteins. Several drugs can block these enzymes and inhibit the generation of bioactive lipid mediators. For example, nonsteroidal anti-inflammatory drugs (NSAIDs), such as indomethacin or ibuprofen, target mainly the COX enzymes, thus prostaglandin biosynthesis, to alleviate inflammation [1–3]. Interestingly, the recognition of the *C. albicans* cell wall β-glucan by dectin-1 on the surface of macrophages enhances macrophage cytosolic phospholipase A2 activity and COX expression, thus promoting prostaglandin biosynthesis in the host [4,5]. Prostaglandin E_2_ (PGE_2_) is known to inhibit the Th1 response and promote Th2 immune responses [6,7]. Following *C. albicans* recognition, the induced PGE_2_ production also enhances Th17 response [8].

Human pathogenic fungi can also produce eicosanoids, mainly PGE_2_, themselves [9]. However, they lack COX and LOX required for their production, which suggests the presence of alternative routes for prostaglandin biosynthesis. Such routes have been identified in *C. albicans* (*FET3* and *OLE2* mediated pathways) [9], *C. neoformans* (*LAC1*) [10] and *Aspergillus* (*PPO* gene family) species [11]. These fungal eicosanoids play a role in fungal pathogenesis, as deletion of *LAC1* in *C. neoformans* resulted in attenuated virulence while disruption of *PPO* genes drastically reduced asexual spore formation in *Aspergillus nidulans* and leads to a hyper-virulent phenotype in *A. fumigatus* [11]. Additionally, the yeast to hyphal transition in *C. albicans* is regulated in part by PGE_2_ and mature biofilms also produce this prostaglandin [12,13].

*C. parapsilosis*, is an important opportunistic human fungal pathogen, which belongs to the CTG clade of the *Ascomycetes* family. After *C. albicans*, it is the second or third leading cause of invasive candidiasis [14]. In particular, low birth weight infants are at increased risk for *C. parapsilosis* infection [15,16]. Previously, we demonstrated that *C. parapsilosis* is also able to produce immunomodulatory prostaglandins from exogenous arachidonic acid. Although, in contrast with *C. albicans*, the fatty acid desaturase *OLE2* is not involved in the process; hence, the molecular mechanisms behind eicosanoid biosynthesis remain to be elucidated [17].

In the current study, our aim was to identify and characterize genes involved in *C. parapsilosis* eicosanoid biosynthesis. Following arachidonic acid induction, we performed RNA sequencing and rigorously investigated gene function through characterizing the generated corresponding deletion mutant strains. We determined that homologues of a multi copper oxidase (*FET3*), an Acyl-CoA thiolase (*POT1*) and an Acyl-CoA oxidase (*POX1–3*) significantly impacted eicosanoid synthesis. Furthermore, according to our results, all three genes influence the virulence of *C. parapsilosis in vitro* and also regulate pathogenicity *in vivo.*

## Results

### Identification of differentially regulated genes following arachidonic acid induction

To identify genes involved in the biosynthesis of lipid mediators, we performed global transcriptomic analysis on *C. parapsilosis* GA1 [18] yeast cells following growth in the presence of arachidonic acid (AA). As a control [zero amount versus background exposure increase (OH)], equal amounts of cells were grown without the addition of arachidonic acid. Altogether, 151 genes showed significantly altered expression (fold change > 1.5) in the presence of arachidonic acid when compared to control. Out of the 151 genes, 68 genes were upregulated, while 83 genes showed decreased expression levels (Table S1). Hierarchical clustering and principal component analysis (PCA) (Figure S1) showed that the three replicates from each group clustered together by condition, confirming the reliability of the obtained results.

**Figure 1.**
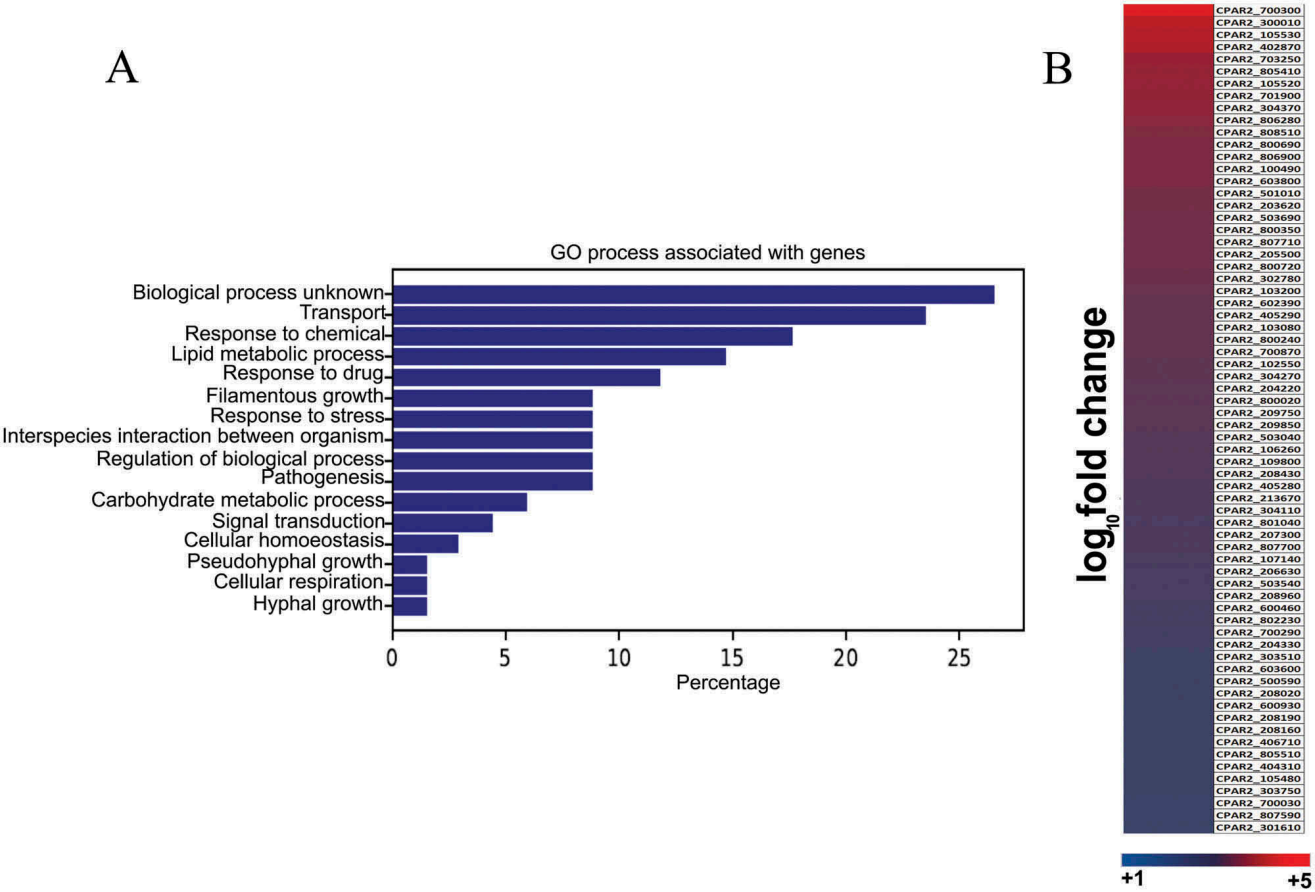
RNA sequencing and data analysis. (a) Global gene expression analysis was performed on the wild type *C. parapsilosis* strain after 3 hours of growth in presence of arachidonic acid. Functional analysis of genome wide expression data suggests that lipid metabolism and transport related pathways are significantly altered in the presence of arachidonic acid. (b) Heat map shows all the *C. parapsilosis* genes upregulated in presence of arachidonic acid. For further information see supplementary table S1.

Functional categorization of the genes by Gene Ontology (GO) term analysis using the candida genome database [19] showed, that 14% of the genes involved in lipid metabolic processes (GO:0006629) are upregulated (Figure 1(a)). The heat map indicates all of the upregulated ORFs in the induced condition (Figure 1(b)). Detailed GO term annotations of the upregulated genes can be found in supplementary Table S1. For further analyzes, we selected six upregulated genes with known lipid metabolic process regulatory homologous in *C. albicans*, (Table 1). Using these genes, we further validated the RNA sequencing data results by performing qRT-PCR analysis (Table 2). qRT-PCR confirmed that the six genes were upregulated following arachidonic acid pretreatment in both *C. parapsilosis* GA1 as well as in a second strain, CLIB 214. As five out of six genes showed higher expression levels in the CLIB 214 type strain, we subsequently used this strain for further analyzes.

**Table 1.** Six up-regulated genes from the RNA sequencing data analysis and their homologues in *C. albicans* and their fold change expression values in *C. parapsilosis.*

CPAR2 GeneID	*Candida albicans* homologue	Fold change
CPAR2_807710	*POX1-3(2)*	2.48
CPAR2_205500	*ECI1*	2.48
CPAR2_102550	*FAA21*	2.19
CPAR2_800020	*POT1*	2.10
CPAR2_807700	*POX1-3(1)*	1.80
CPAR2_603600	*FET3*	1.63

**Table 2.** Confirmation of the RNA sequencing data: fold change values of the 6 selected genes in both *C. parapsilosis* strains determined by qRT-PCR analysis.

CPAR GeneID	CLIB	GA1
CPAR2_807710	20.96	4.70
CPAR2_205500	7.27	2.41
CPAR2_102550	11.81	3.14
CPAR2_800020	3.63	2.17
CPAR2_807700	7.48	1.08
CPAR2_603600	0.67	3.21

In order to determine the role of the identified genes in eicosanoid biosynthesis, we generated homozygous deletion mutant strains for each candidate gene by applying a gene disruption method previously introduced by Holland *et al* [20].

### Homozygous deletion mutants of CPAR2_603600, CPAR2_800020 and CPAR2_807710 genes showed a significant reduction in extracellular lipid mediator production

Using liquid chromatography-mass spectrometry (LC/MS), we analyzed all null mutant strains for their ability to produce eicosanoids. This approach has previously demonstrated that the storage of arachidonic acid results in the production of auto-oxidation products [21,22].

Therefore, we also incubated 100 µM arachidonic acid in PBS at 30°C for 24 hours to measure the amount of spontaneously produced eicosanoids without the presence of fungal cells.

**Figure 2.**
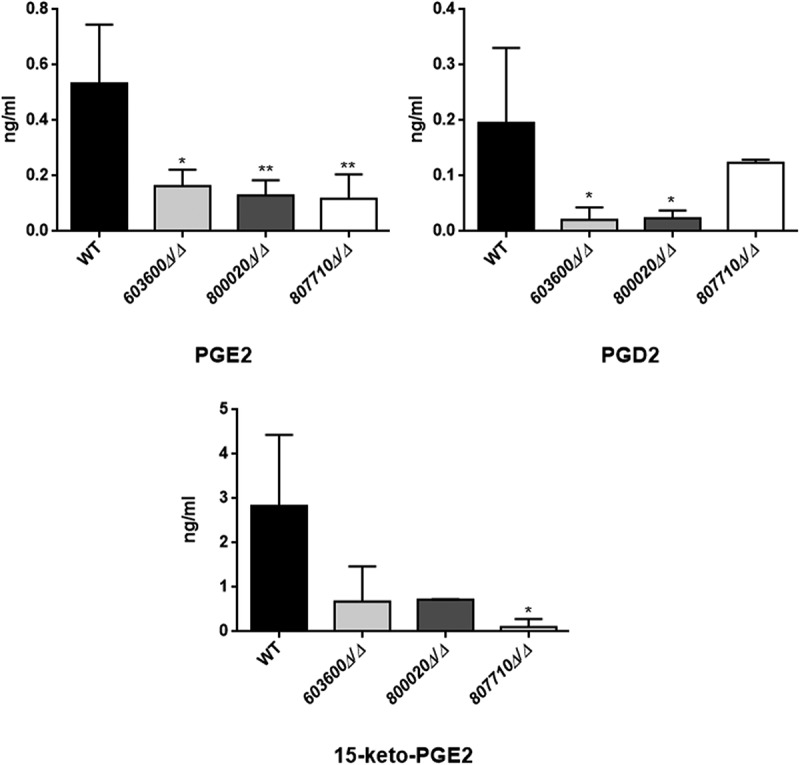
Reduced eicosanoid production by *C. parapsilosis* mutant strains. *C. parapsilosis* CLIB 214 wild type strain and three null mutant strains *603600Δ/Δ, 800020Δ/Δ* and *807710Δ/Δ* were grown for 24 hours at 30°C in the presence of 100 μM arachidonic acid in PBS. PBS with only arachidonic acid served as control. After sterile filtration, 100 µl of cell-free samples were analyzed with LC/MS. Among the examined eicosanoids, the three null mutants showed significant reductions in PGE_2_ (*CPAR2_603600Δ/Δ, CPAR2_800020Δ/Δ* and *CPAR2_807710Δ/Δ*) and one strain in 15-keto-PGE_2_ (*CPAR2_807710∆/∆)* production. Strains *603600∆/∆* and *800020∆/∆* had a strong trend toward a lower production of PGD_2_ compared to the wild type strain. *P < 0.05, **P < 0.01.

The LC/MS data for the secretory eicosanoid analysis revealed that the deletion mutant strains of *CPAR2_603600, CPAR2_800020* and *CPAR2_807710* produced less prostaglandin D_2_ (PGD_2_), although in case of *CPAR2_807710* this reduction was not significant. Further, all three mutant strains showed a significant decrease in PGE_2,_ production. In contrast, a reduction in 15-keto-prostaglandin E_2_ (15-keto-PGE_2_) production was measurable only in case of *CPAR2_807710* (Figure 2). *CPAR2_102550Δ/Δ, CPAR2_205500Δ/Δ* and *CPAR2_807700Δ/Δ* did not show any difference in PGD_2_, PGE_2_ and 15-keto-PGE_2_ production (Figure S2).

### Fungal 5-D2-isoprostane identified during LC/MS analysis

**Figure 3.**
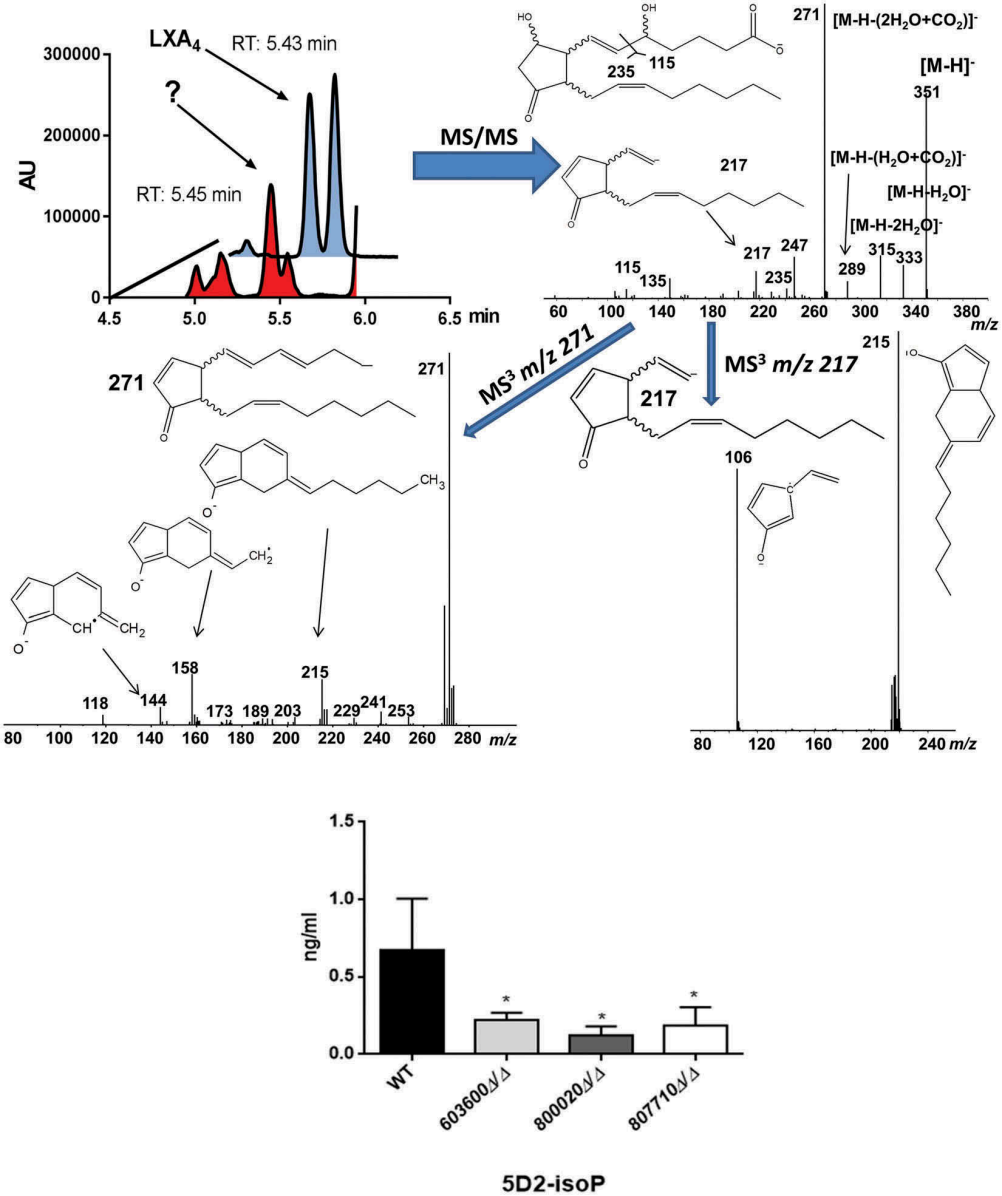
Suggestive identification and measurement of fungal 5D2-IsoP in wild type and eicosanoid mutant strains. Upper left, LC/MS analysis of cell supernatant after growing *C. parapsilosis* wild type strain in presence of 100 μM arachidonic acid in PBS. Blue trace shows the elution of an authentic LXA_4_ standard (1 ng/mL) in the transition *m/z* 351-> 115. Red trace shows the elution of an overlapping signal in a representative *C. parapsilosis* sample. (Upper right) MS/MS spectrum of the signal co-eluting with LXA_4_ and chemical structure of the candidate molecule 5D2-IsoP. Middle MS^3^ spectra of *m/z* 271 (left) and *m/z* 217 (right). Lower panel, *CPAR2_800020Δ/Δ* showed a significant reduction in 5D2-IsoP production, although 5D2-IsoP levels also decreased in *CPAR2_603600Δ/Δ* and *CPAR2_807710Δ/Δ* strains.

The LC/MS data for the secreted eicosanoids also revealed, that *C. parapsilosis* incubations gave rise to an uncommon isoprostane likely identified to be a 5-D2-IsoProstane which is considered an autoxidative isomer of PGD_2_ (Figure 3). Investigating the selective ion trace *m/z* 351 -> 115 and comparing retention times (RT) and tandem mass spectra we observed a signal which overlapped with an authentic standard of lipoxin A4 (LXA_4_), however, some differences in the fragment intensities during tandem mass spectrometry prompted us to further evaluate the identity of this signal. We used a secondary solvent system for confirmatory analysis (data not shown). This analysis revealed that the signal suspected to be LXA_4_ was indeed something else as authentic standard and analyte no longer co-eluted. In order to propose a possible structure for the obtained signal we carried out tandem mass spectrometry as well as MS^3^ analysis of the obtained peak. As can be seen from Figure 3, based on the mass spectrometric data, we propose the observed oxylipin to be a 5-D2-IsoP [23]. The fragment *m/z* 115 is indicative of a 5-hydroxy group, in combination with the MS/MS fragments m/z 217 and 271 as well as the MS^3^ fragments *m/z* 215 and 106 we propose 5-D2-IsoP to be a likely candidate molecule for the observed signal. According to our data, besides the low production of prostaglandins, mutant strains incubations of *CPAR2_603600, CPAR2_800020* and *CPAR2_807710* also contained less 5-D2-IsoP compared to the wild type (Figure 3). This reduction was significant in all the mutant strains. No significant differences were found in case of *CPAR2_102550Δ/Δ, CPAR2_205500Δ/Δ* and *CPAR2_807700Δ/Δ* in terms of 5-D2-IsoP (Figure S2).

### *Phagocytosis and killing of* 603600∆/∆, 800020∆/∆ *and* 807710∆/∆ *mutants by human macrophages*

**Figure 4.**
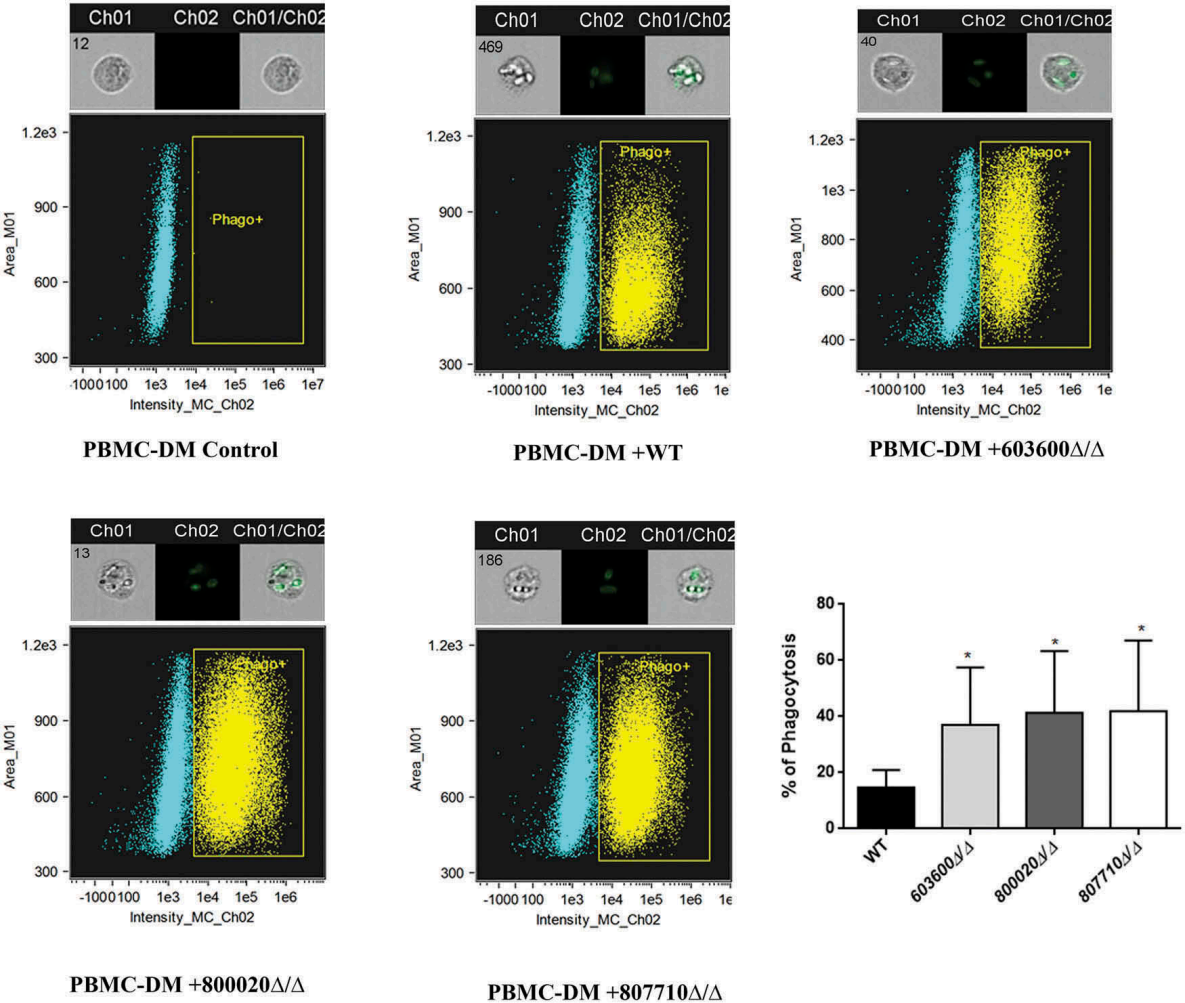
Phagocytosis of wild type and eicosanoid mutant strains by human macrophages by imaging flow cytometry. The phagocytosis of wild type and *C. parapsilosis* eicosanoid mutant cells by PBMC-DMs was analyzed by an imaging flow cytometer. Yeast cells were labeled with AlexaFluor447 and co-incubated with macrophages for 2 hours at 37°C with 5% CO_2_. Data were obtained from three independent experiments. Representative dot plots and summarized data of the flow cytometric analysis are shown. *P < 0.05.

**Figure 5.**
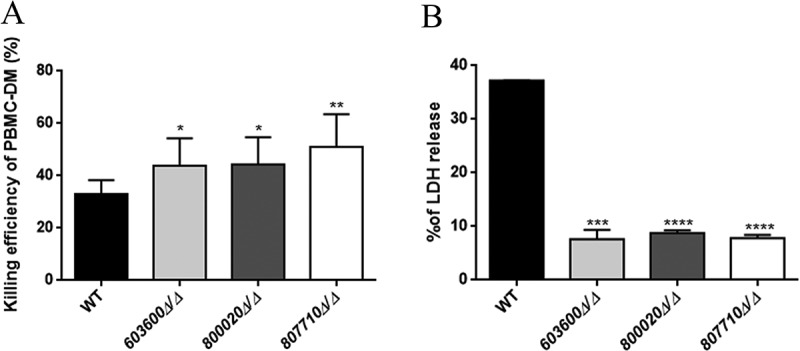
Killing of *C. parapsilosis* strains by human PBMC-DMs and host cell damage. (a) The efficiency of killing by human macrophages was analyzed by CFU determination. Experiments were performed in triplicates. The obtained data represents the killing efficiency of macrophages gained from 5 healthy donors. (b) Human PBMC-DMs were infected with wild type and *C. parapsilosis* eicosanoid mutants for 24 hours and LDH release was measured. LDH release is expressed as % of positive control. *P < 0.05, **P < 0.01, ***p < 0.002, ****p < 0.0001.

Human peripheral blood monocyte derived macrophages (PBMC-DM) were used to characterize the virulence properties of mutant strains with altered eicosanoid producing profiles. We first examined the phagocytic activity of PBMC-DM by fluorescence-activated cell sorting (FACS). *Candida* yeast cells were labeled with the fluorescent dye Alexa Fluor 488 (a succimidyl ester) and then co-incubated with PBMC-DMs for 2 hours. Our results indicated that PBMC-DMs ingested each of the mutant strains more efficiently compared to the wild type strain (Figure 4). We also examined the yeast cell killing efficiency of PBMC-DMs by comparing the recovered fungal CFUs. Our data showed that each of the mutant strains were killed more effectively by PBMC-DMs in comparison to the wild type strain (Figure 5(a)).

### *Host cell damage is decreased by* 603600∆/∆, 800020∆/∆ *and* 807710∆/∆ *strains*

Next, we examined the mutant strains’ abilities to cause host cell damage by measuring the amount of LDH released by human PBMC-DMs following infection. As shown in Figure 5(b), we found that the PBMC-DMs showed significantly lower LDH release when infected with the mutants compared to the wild type. Our results indicated that *603600∆/∆, 800020∆/∆ and 807710∆/∆* mutant strains show a lower host cell damaging capacity than wild type cells (Figure 5(b)).

### *Macrophages favor the uptake of* 603600∆/∆, *800020∆/∆ and 807710∆/∆ strains over the wild type*

**Figure 6.**
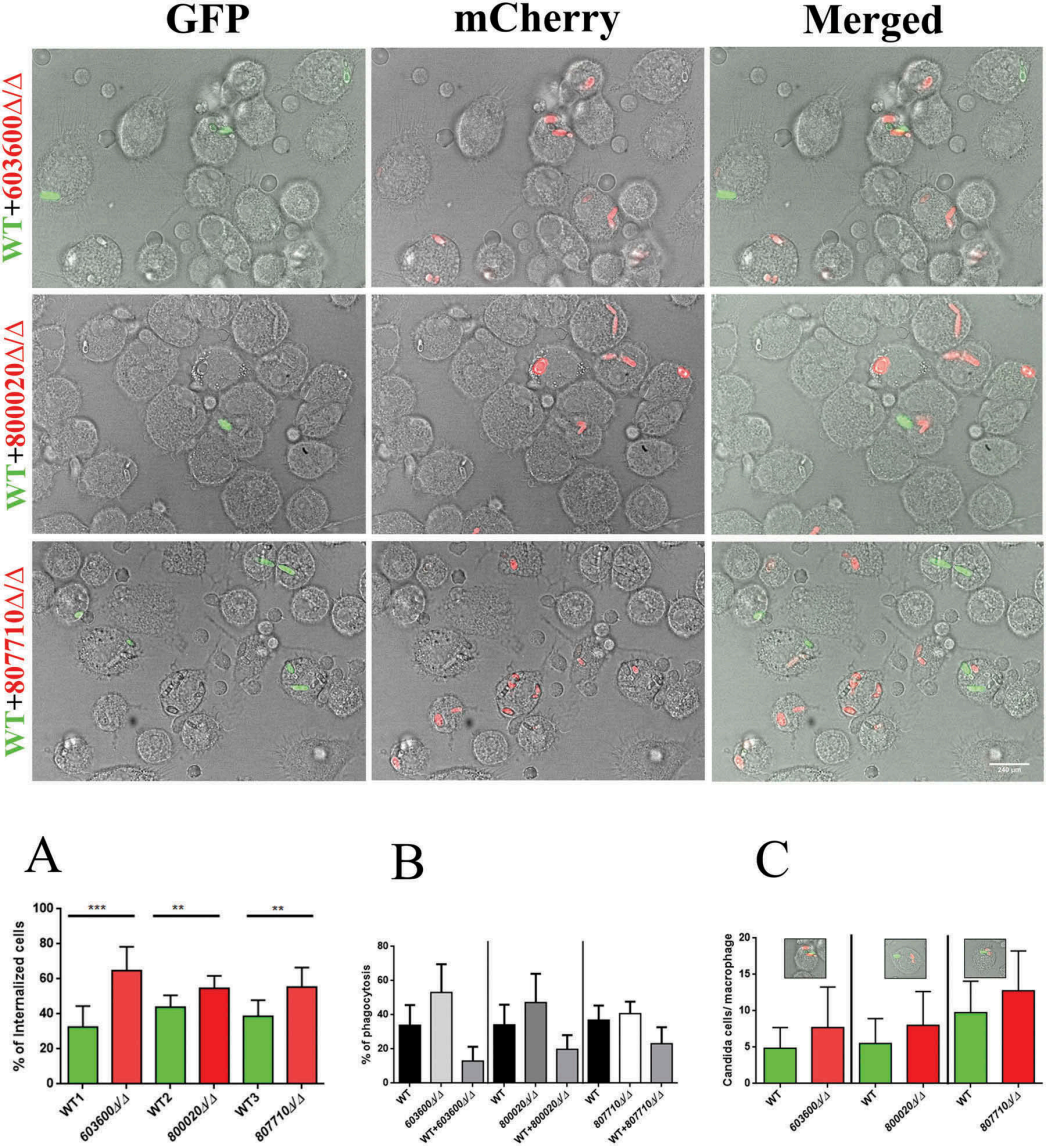
Phagocytic competition assay. The competition assays were performed using combinations of GFP tagged wild type *C. parapsilosis* and mCherry tagged eicosanoid mutant strains. Equal numbers of yeast cells expressing GFP or mCherry were mixed together and added to human PBMC-DMs at the MOI of 1:2. (a) Significantly higher number of mutant cells (red) were internalized compared to the wild type cells (green). (b) Percentage of phagocytic macrophages in each case (c) Number of macrophages with both wild type and mutant strain in each case did not show any difference. **P < 0.01, ***p < 0.002.

We tested the uptake efficiency of the mutants by human PBMC-DMs using a competition assay and compared them to the wild type strain. During the assay, we used differently labeled *Candida* strains: a GFP tagged wild type strain and mCherry labeled mutant strains. PBMC-DMs were infected with 2 types of fungal cells mixed in a ratio of 1:1 and co-incubated for 2 hours. Interactions were monitored via fluorescent imaging. The percentage of internalized cells was calculated for each strain, and the values for the mutants were compared to those of the wild type’s (Figure 6(a)). We calculated the percentage of phagocytosis by counting the total number of PBMC-DMs (Figure 6(b)). The number of green and red cells inside a single PBMC-DM which phagocytosed both type of cells was also analyzed (Figure 6(c)). Overall our results revealed that, PBMC-DMs significantly preferred the uptake of *603600∆/∆, 800020∆/∆* and *807710∆/∆* cells over the wild type. However, when the PBMC-DMs phagocytosed both type of cells we did not find any significant preference for the mutant cells.

### Influence on phagosome-lysosome fusion

**Figure 7.**
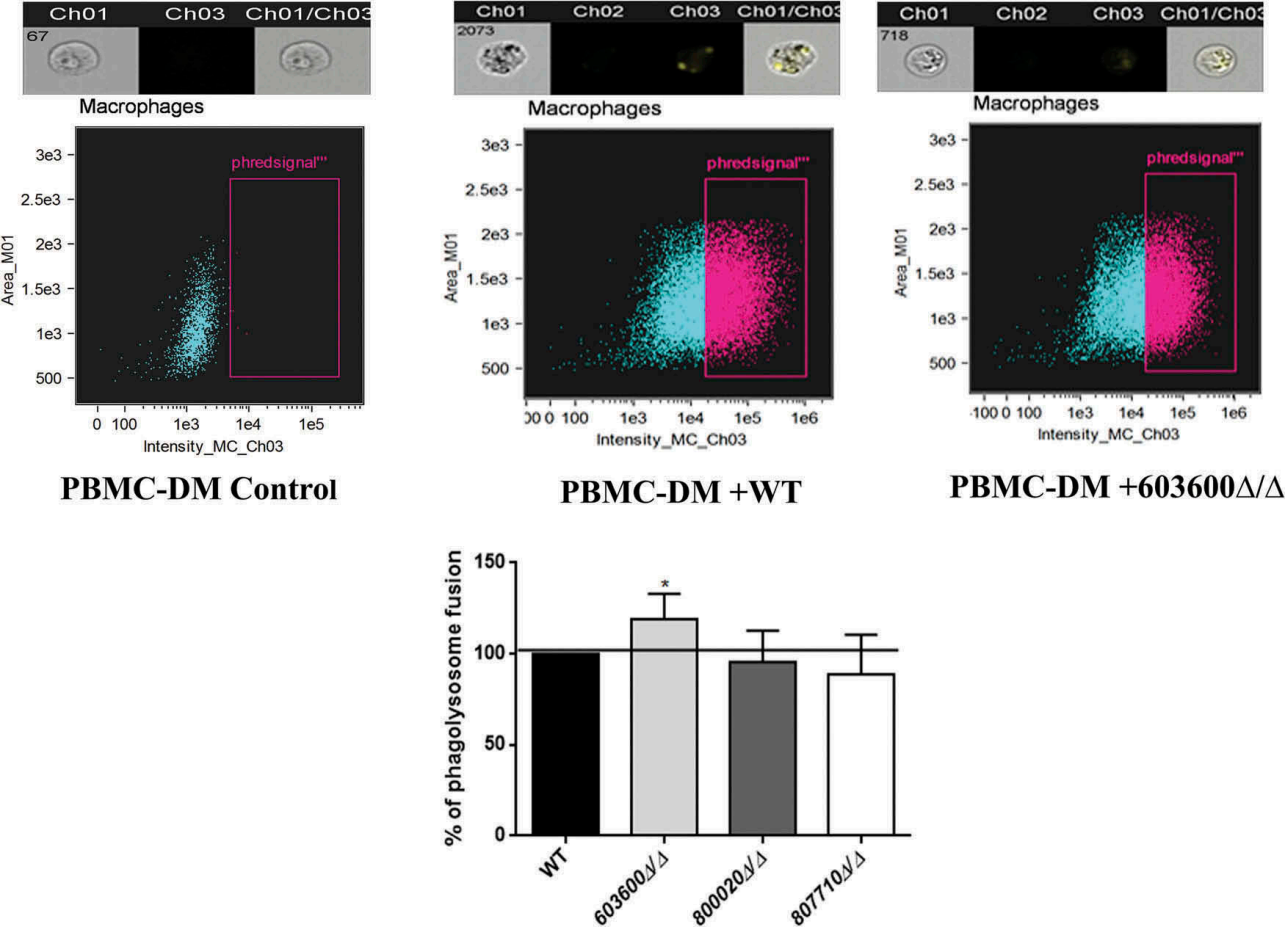
Phagosome-lysosome fusion in response to wild type and eicosanoid mutants. Phago-lysosome co-localization in human PBMC-DMs following the phagocytosis of pHrodo labeled *Candida* cells. Representative picture of a phagocytosing macrophage during quantitative imaging analysis. Ch1: brightfield image, Ch3: green fluorescence channel. Graph showing the difference between the extent of phago-lysosome fusion in case of the wild type and the mutants. *P < 0.05.

Next, we addressed whether the lack of the examined lipid mediators had any effect on phagosome-lysosome fusion in human PBMC-DMs. We analyzed the phagosome-lysosome co-localization after co-incubating pHrodo stained *Candida* cells with PBMC-DMs for 2 hours. Although, all three mutants are phagocytosed and killed more efficiently by PBMC-DMs, only the *603600∆/∆* strain induced a higher rate of phagosome-lysosome fusion, suggesting the corresponding gene’s influence on phagosome maturation (Figure 7).

### Reduction in prostaglandin production alters the cytokine response

Eicosanoid derived lipid mediators effectively regulate inflammatory responses. Multiple functions, dependent on concentration, location and timing have been described for the prostaglandins (e.g.PGD_2_ and PGE_2_) [1-3,24,25]. In order to examine the immunological responses triggered by the *603600∆/∆, 800020∆/∆* and *807710∆/∆*, we stimulated human PBMC-DMs for 24 hours with each strain and determined the amount of cytokine and chemokine production. During the experiments we measured pro-IL1β, TNFα, Interleukin-1 receptor antagonist (IL-1ra), interleukin-6 (IL-6), interleukin-8 (IL-8) and interleukin-10 (IL-10) levels.

**Figure 8.**
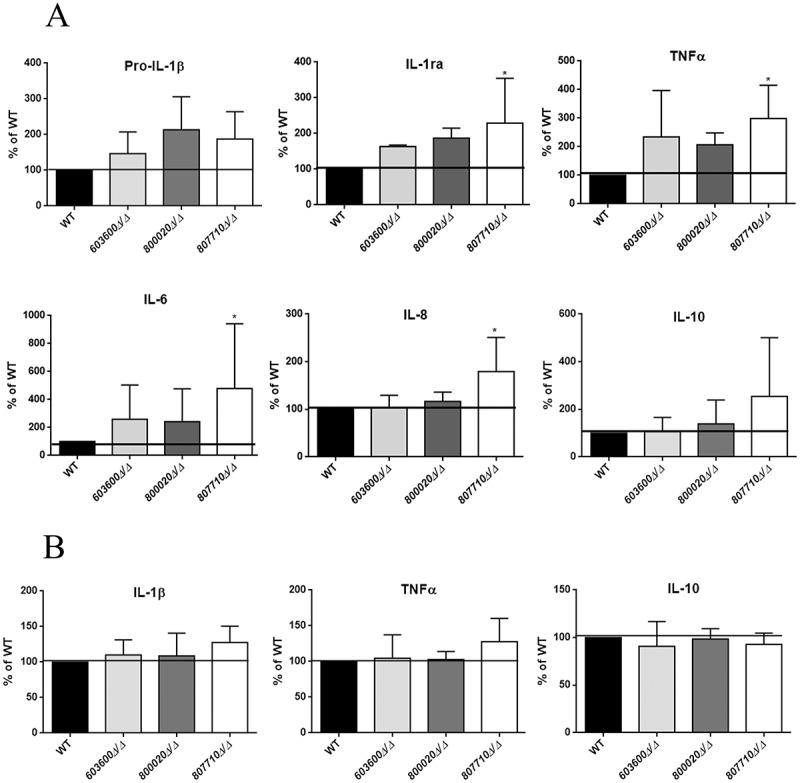
Cytokine secretion of human macrophages in response to wild type and eicosanoid mutants. (a) Pro IL-1β, IL-1ra, TNFα, IL-6, IL-8, and IL-10 levels were measured by ELISA after stimulation of PBMC-DMs with wild type or eicosanoid mutant strains for 24 hours. Data were normalized for each donor to cytokine levels induced by the wild type strain (100%) to minimize donor to-donor variability. (b) IL-1β, TNFα and IL-10 were also measured after infection of human PBMCs with the same strains. Data represent % cytokine production ± SEM for 6 donors. *P < 0.05.

PBMC-DMs infected with *807710∆/∆* produced significantly higher amounts of the pro-inflammatory cytokine, Il-6, TNFα, chemokine IL-8 and IL-1ra levels compared to the wild type strain. Similarly, PBMC-DMs stimulated with *603600∆/∆* and *800020∆/∆* produced higher amounts of Pro-IL-1β, IL-1ra, Il-6 and TNFα compared to the reference strain, although these differences were not significant. Further, these two mutant strains did not induce IL-8 secretion. In terms of IL-10 production, none of the mutant strains showed a significant difference compared to the wild type (p > 0.05), however, *807710∆/∆* showed a trend towards higher levels of release (Figure 8(a)).

We analyzed the cytokine production by human primary PBMCs following fungal stimuli. PBMCs infected with *807710∆/∆* showed a trend towards a higher IL-1β and TNFα (Figure 8(b)) release, although there was no difference in case of the other mutant strains. These data suggest that fungal eicosanoids may also influence the host cytokine response.

### 603600∆/∆, 800020∆/∆ *and* 807710∆/∆ *strains show attenuated virulence in vivo*

**Figure 9.**
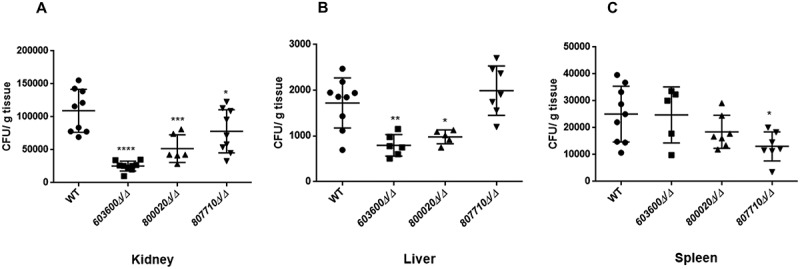
Fungal burden in organs after intravenous infection. Five mice were infected intravenously with 2 × 10^7^/100 µl of *C. parapsilosis* wild type or eicosanoid mutant cells. CFUs recovered from kidney (a), liver (b), and spleen (c) after 3 days of the infection. The results are pooled data from two independent experiments. *P < 0.05, **P < 0.01, ***p < 0.002, ****p < 0.0001.

Following *in vitro* studies, we also aimed to examine the virulence of the *603600∆/∆, 800020∆/∆* and *807710∆/∆ in vivo* using a mouse model of disseminated candidiasis. Following the intravenous infection of BALB/c mice, the fungal burdens of different organs were determined three days after the infection. After CFU recovery, we found that mice infected with *603600∆/∆* showed significantly reduced fungal burdens in the liver and kidneys, while *800020∆/∆* inoculated mice also revealed lower fungal burdens in the liver and kidneys. Finally, CFUs recovered following *807710∆/∆* injection were significantly less in the spleen and kidneys compared to those recovered from wild type-infected mice (Figure 9). These results indicate that these eicosanoid biosynthesis genes play role in *C. parapsilosis* virulence.

## Discussion

Eicosanoids, a group of bioactive mediators, are signaling molecules with diverse physiological and pathological functions known to modulate inflammatory responses. Prostaglandins, leukotrienes and lipoxins are groups of eicosanoids involved in pro-, and/or anti-inflammatory responses. During vasodilation prostaglandins and leukotrienes induce increased permeability of post capillary venules and also recruit complement components and leukocytes to the site of inflammation [2]. In contrast, lipoxins act as pro-resolving lipid mediators, as for example, LXA_4_ inhibits the recruitment of neutrophil and eosinophil granulocytes in post capillary venules [3]. Previously, it has been hypothesized that, during fungal infection the invading fungi induce host prostaglandin biosynthesis that causes a local anti-inflammatory response, which in turn could contribute to fungal invasion [26].

In recent years, several studies have revealed that human pathogenic fungi are able to produce lipid mediators, specifically prostaglandins that might as well contribute to their virulence. This group of pathogens includes species such as *A. nidulans, A. fumigatus, C. neoformans, C. albicans* and *C. parapsilosis.*

Investigations have revealed several biosynthetic pathways used by some of these pathogens. As an example, *A. nidulans* and *A. fumigatus* have three dioxygenase-encoding genes, namely *ppoA, ppoB* and *ppoC* with high sequence similarity to mammalian cyclooxygenases [11]. Besides regulating sexual and asexual sporulation, all three *ppo* genes contribute to prostaglandin biosynthesis, possibly via oxygenating arachidonic acid, thereby generating the prostaglandin precursor PGH_2_ [11].

Other studies have revealed that certain pathogenic fungi, such as *C. albicans* and *C. neoformans*, do not possess COX-like enzymes, thus they must have evolved prostaglandin biosynthetic pathways different from those of mammals [12,13]. In *C. neoformans*, a member of the multicopper oxidase family, the Lac1 laccase regulates PGE_2_ biosynthesis, possibly by converting the prostaglandin precursor PGG_2_ to PGE_2_ and15-keto-PGE_2_ [27].

In *C. albicans* however, the fatty acid desaturase *OLE2* and a multicopper oxidase *FET3* play a role in prostaglandin production via novel pathways using exogenous arachidonic acid as precursor [9]. *CaFET3*, a laccase homolog (and also a member of the Fet family of multicopper oxidases) is suggested to regulate PGE_2_ biosynthesis through a mechanism similar to that of *LAC1* in *C. neoformans*. On the other hand, Ole2, a putative delta9 desaturase also containing a cytochrome B domain, is hypothesized to regulate PGE_2_ production via the oxidation of exogenous arachidonic acids [9].

Notably, in these species, the identified genes are pleiotropic regulators as they also participate in mechanisms such as sporulation (*ppo* genes), cell wall homeostasis (*LAC1*) or iron uptake (*FET3*), and thus contribute to virulence in a complex manner. Nevertheless, the role of fungal prostaglandins in virulence is evident as, in all yet examined fungal species, their production has been associated with markedly altered immune responses [11,26]. To expand our knowledge about the role of fungal eicosanoids in pathogenesis, we examined lipid mediator production in another important human fungal pathogen, *C. parapsilosis*. As the incidence of this species has increased over the past two decades and the patient group at risk includes immunosuppressed children and adults as well as neonates, understanding the pathogenesis of *C. parapsilosis* has gained increased attention [16,28]. Preliminary studies have started to elucidate the prostaglandin profile of this species [17], however, the involved biosynthetic pathways and the presence or role of other fungal eicosanoids remained elusive. Previously, we have shown that in the presence of exogenous arachidonic acid, *C. parapsilosis* is capable of producing fungal prostaglandins, although *OLE2* is not involved in the synthetic mechanisms, leaving the corresponding biosynthetic processes unexplored [17].

Therefore, in the current study, we aimed to reveal regulators involved in eicosanoid biosynthetic mechanisms and to investigate their roles in the virulence of this species. Following arachidonic acid induction, we identified three genes that significantly influence the biosynthesis of fungal prostaglandins. Our results indicate, that *CPAR2*_*603600*, a homologous gene of *CaFET3* is involved in PGE_2_ and PGD_2_ production, *CPAR2_807710*, a homologue of the acyl-coenzyme A oxidase, *ScPOX1-3*, regulates PGE_2_ and 15-keto-PGE_2_ synthesis, and *CPAR2*_*800020*, a homologue of 3-ketoacyl-CoA thiolase, *ScPOT1*, influences PGE_2_ biosynthesis. Using LC/MS analysis, we observed that the disruption of each gene led to a decrease in the corresponding eicosanoids’ production.

While *CaFET3* is known to interfere with fungal prostaglandin production [9], no such role has been associated with *CaPOX1-3*, and *CaPOT1* in *C. albicans*, suggesting a novel function of the corresponding homologues in *C. parapsilosis*.

The roles of *CaFET3* and *CaPOT1* in *C. albicans’* virulence have been investigated. Deletion of *CaFET3* resulted in reduced adhesiveness to fibroblasts, although no significant differences were observed between the virulence of the wild type and the *Δ/Δfet3* strain in a mouse model of systemic candidiasis [29]. In contrast with *C. albicans*, upon the removal of the ortholog of *FET3* in *C. parapsilosis* indeed influenced the fungi’s virulence in vivo, given the lower amount of fungal burden measured. In contrast, *CaPOT1*, a 3-ketoacyl-CoA thiolase, involved in fatty acid utilization, is not required for virulence in an embryonated chicken egg infection model [30]. However, its ortholog in *C parapsilosis* is involved in virulence regulation as represented by both our *in vitro* and *in vivo* virulence studies. Unfortunately, to date, we lack information about the hypothetical acyl-coenzyme A oxidase’s (*CaPOX1-3*) role in *C. albicans* pathogenesis. Interestingly, our results also suggest the involvement of the corresponding gene’s ortholog in the virulence of *C. parapsilosis*.

These data suggest, that in contrast to *C. albicans*, the homologous genes of *FET3, POT1* and *POX1-3* in *C. parapsilosis* indeed contribute to fungal virulence, although the corresponding mechanisms still need to be elucidated.

Interestingly, *CPAR2_603600* might be involved in delaying phagosome-lysosome fusion, a phenomenon not yet investigated in *C. albicans*, although additional studies are needed to confirm this mechanism hypothesis.

Fungal prostaglandins produced by *C. albicans* and *C. neoformans* alter host cytokine responses by down-regulating chemokine (IL-8) and pro-inflammatory cytokine (e.g. TNFα) production while concomitantly up-regulating anti-inflammatory responses via promoting IL-10 release [26]. Our results suggest a similar effect with *C. parapsilosis* eicosanoids, as mutant strains defective in prostaglandin production induced higher pro-inflammatory cytokine responses, as shown by the increased levels of Pro-IL-1β, IL-6 and TNFα released by human PBMC-DMs. Although, stimulation of human PBMCs did not result in a significant increase of the examined cytokines in case of any of the mutant strains. These data suggest that *CPAR2_807710, CPAR2_800020* and *CPAR2_603600* contribute unequally to the alteration of host immune responses.

This is the first study reporting the presence of 5D2-IsoP in fungal incubations that might actively contribute to fungal virulence. We have identified three *C. parapsilosis* eicosanoid biosynthesis regulatory genes, namely *CPAR2_807710, CPAR2_800020* and *CPAR2*_*603600*, that are involved in the production of fungal prostaglandins. Virulence studies performed with the corresponding null mutant strains suggests that these regulatory genes also influence the fungi’s virulence. Although, further investigation is needed to thoroughly understand the importance of fungal eicosanoids, our results can contribute to a better understanding of host pathogen interactions during candidiasis.

## Materials and methods

### Strains and growth conditions

All *C. parapsilosis* strains used in this study are listed in Table S2. Strains were grown in YPD (1% dextrose, 1% peptone and 0.5% yeast extract) at 30°C. For colony selection 2% agar was added in the media. Nourseothricin (NAT) resistant transformants were selected on YPD plates supplemented with 100 μg/ml NAT. Cells transformed with *LEU2* and *HIS1* markers were selected on synthetic complete media (SC; 2% Dextrose, 0.95% yeast nitrogen base, mixture of amino acid, 2% agar) without leucine and histidine.

### RNA extraction

For RNA sequencing, the *C. parapsilosis* GA1 strain was grown overnight in 2 ml of YPD media at 30°C with continuous shaking applied at 180 rpm. The next day, cells were washed 3 times with PBS and then counted using a Burker’s chamber. Cell concentration was adjusted to 2 × 10^7^ cells per 10 ml PBS supplemented with 500 μM of arachidonic acid in triplicates. As a control, cells were also grown in 10 ml PBS supplemented with ethanol only, as it is the dissolving agent used for arachidonic acid solubilization. After 3 hours of growth at 30°C, RNA was isolated using the Ribopure Yeast RNA isolation Kit (Ambion) following the manufacturer’s instructions. For validating the RNA sequencing data, *C. parapsilosis* CLIB and GA1 cells were grown as described above and RNA extraction was performed using the same kit.

### RNA sequencing and data analysis

RNA-seq library preparation and sequencing, paired-end reads (Illumina Truseq V2 PolyA, not -stranded, V3-150, 2 × 75bp, 25M read) were generated from three biological replicates of induced and non-induced *C. parapsilosis* cells.

For initial quality assessment and data preprocessing we used FastQC 0.10.1 [http://www.bioinformatics.bbsrc.ac.uk/projects/fastqc/] quality assessment and Trimmomatic v0.32 [31] where preset conditions were applied for quality cutoff to remove low quality regions from the raw data [parameters used LEADING:3 TRAILING:3 SLIDINGWINDOW:4:15 MINLEN:36.]. Mapping was carried out against the reference genome obtained from the Candida Genome Database [19]. We used the splice junction sensitive mapper Tophat 2.01.13 [32] with default settings, and mapped by applying the bowtie 2.2.4 [33] short read mapper. The counts per gene were estimated by using flux-capacitor [34]. To estimate differential expression, we used the R package Deseq2 [35] with a cutoff of log2fold change of 1.5. Gene Onthology enrichment was performed using the CGD database [19].

### Reverse transcription PCR

A total of 500 ng RNA was used for cDNA synthesis. The cDNA was synthesized using the Revert Aid first Strand cDNA Synthesis Kit (Thermo Scientific) according to the manufacturer’s instructions.

### Real time PCR

Real time PCR was performed in a final volume of 20 μl using Maxima SYBR Green/Fluorescein qPCR Master Mix (2X) (Thermo Scientific). The reaction was performed in C1000 Thermal Cycler (Bio-Rad) using the following protocol: 95°C for 3mins, 95°C for 10 s, 60°C for 30 s, 65°C for 5 s for 50 cycles. Fold change in mRNA expression was calculated by ΔΔCt method (Real–Time PCR applications guide BIO-Rad). *TUB4* gene was used as a housekeeping gene for an internal control.

### Preparation and transformation of competent cells

All the deletion mutants and fluorescently labeled strains were constructed by chemical transformation. The parental CLIB 214 double auxotrophic strain (CPL2H1) [20] and the mutants were inoculated in 2 ml YPD and grown overnight at 30°C with shaking applied at 180 rpm. Then the culture was diluted to an OD_600_ of 0.2 in 30 ml YPD broth and grown at 30°C to an OD_600_ of 0.8–1. The culture was centrifuged at 4000 rpm for 5 min and the pellet was suspended in 3 ml of ice-cold water. The cells were again centrifuged at 4000 rpm for 5 min and washed with 1 ml ice cold TE-LiOAC (0.1M lithium acetate, 10 mM TRIS and 1mM EDTA). After centrifugation the final pellet was dissolved in 300 μl of ice cold TE-LiOAC. A transformation mix was prepared with 100 μl competent cells, 10 μl single stranded salmon sperm DNA (10 mg/ml) and 25–30 μl fusion PCR product and the mixture was kept at 30°C for 30 min. Finally, 700 μl PLATE (0.1M lithium acetate, 10 mM tris, 1mM EDTA and 40% PEG 3350) was added to the transformation mix and cells were kept overnight at 30°C. The next day, cells were heat shocked at 44°C for 15 min, centrifuged and washed with 1 ml of YPD media. The cells were then re-suspended in 1 ml of YPD and incubated at 30°C at 200 rpm for 2–3 hours. After the incubation, the cells were centrifuged and dissolved in 100 μl of YPD, then plated on the respective agar plates.

### *Generation of* C. parapsilosis *mutant strains*

For gene deletion, the fusion PCR technique was used, as described previously by Holland et al [20]. Target genes are listed in Table 2 (Figure 1) and the primers are used listed in TabS3. Approximately 500bp upstream and 500bp downstream sequences of the target genes were amplified with primer pairs 1,3 and 4,6 using DreamTaq polymerase (Thermo Scientific). Selection markers *LEU2* and *HIS1* were amplified with primer pairs 2 and 6 from the plasmids pSN40 (*LEU2*) and pSN52 (*HIS1*). Finally, all the PCR products were purified using the PCR Purification kit (Quiagen) and fused with Phusion Taq polymerase (Thermo Scientific) using primers 1and 6. The resulting disruption cassette was transformed into the double auxotrophic CLIB 214 strain.

### Construction of fluorescent tagged strains

All the *C. parapsilosis* fluorescently labeled strains were generated using the Gateway® cloning technology (Invitrogen). The fluorescent constructs were expressed under an overexpression promoter targeted in the *NEUT5* locus. The fluorescence expression was confirmed after checking the strains under fluorescent microscope.

### Sample preparation for secreted eicosanoid measurement

For eicosanoid measurement, 2 × 10^7^ *Candida* cells were inoculated in 10 ml of PBS+ 100 μM arachidonic acid in triplicates and incubated at 30°C for 24 hours. Three tubes with only PBS and 100 μM arachidonic acid were also incubated and served as control. For secreted eicosanoids, the cell supernatant was collected using sterile filtration after centrifugation. 100 µl sample was complemented with 96 µl MeOH and 4 µl internal standard solution (final concentration: 1 ng/ml PGE_2_-d4, LTB_4_-d4 and 15-HETE-d8 and 10 ng/ml DHA-d5) and analyzed without further processing.

### Analysis of lipid mediators by LC/MS

Eicosanoids were analyzed using a targeted LC-MS/MS method according to published protocols with a minor modification [36]. The drying temperature was set to 450°C instead of 400°C. Table S4 shows the MRM characteristics of the monitored eicosanoids. When concentrations were determined external calibration was carried out with linear regression using a weighing factor of *1/x^2^*. For calculating the actual concentration of the secreted eicosanoids, the amount of spontaneously generated eicosanoids (PBS+ AA control sample) were subtracted from the total amount of eicosanoids produced by each strain (data not shown). MS^3^ analysis were carried out under identical conditions using an excitation energy (AF2 value) of either 0.5 or 0.8 (V) as collisional energy in the MS^3^ experiments, investigating the MS/MS fragments *m/z* 217 and 271.

### Isolation and differentiation of PBMCs

Human PBMCs were isolated from buffy coats of healthy individual by density gradient centrifugation [37]. Briefly, blood samples were first diluted with PBS (1:1 dilution). The diluted blood was overlaid above the Ficoll solution (Ficoll Paque PLUS-GE Healthcare) and then centrifuged at 900 rpm for 30 mins. After centrifugation, the mononuclear cell layer was aspirated with a pipette, collected in a separate 50 ml centrifuge tube and then washed 3 times with PBS. Finally, cells were suspended in RPMI 1640 medium (Lonza) supplemented with 100 U/ml penicillin and 100 mg/ml streptomycin. Cells were counted and the concentration was adjusted to 5 × 10^6^ cells/ml. For later experiments, 100 μl of 5 × 10^5^ cells in RPMI were added to U-bottom 96-well plates. For macrophage differentiation, cells were added to flat bottom 24-well plates with the concentration of 1ml of 1 × 10^7^ cells per well and incubated for 90 mins at 37°C in the presence of 5% CO_2_ and 100% humidity. After the incubation, RPMI was removed and cells were washed with 1 ml of PBS. Finally, the cells were re-suspended in X-VIVO 15 media (Lonza) supplemented with 100 U/ml penicillin, 100 mg/ml streptomycin and 10ng/ml recombinant human granulocyte-macrophage colony-stimulating factor (GM-CSF, Sigma-Aldrich). The media was changed every 2^nd^ day for 7 days. During the study, PBMCs were used for cytokine measurement only.

### Infection of PBMCs and PBMC-DMs

For the infection experiments, *C. parapsilosis* strains were grown overnight in 2 ml YPD media, washed 3 times with PBS and adjusted to the proper concentrations used for each experiment. For the infection of both human PBMCs (cytokine measurement only) and PBMC-derived macrophages, the multiplicity of infection (MOI) was 1:5. *Candida* cells were dissolved in 100 μl of either RPMI/PS (PBMC) or GM-CSF-free X-VIVO 15 (PBMC-DM) medium and were added to host cell containing wells.

### Killing assay

Human PBMC-DMs (5 × 10^5^/well) were co-incubated with *C. parapsilosis* cells in 24-well plastic culture plates at a MOI of 1:5. As a control, equal amounts of *Candida* cells were incubated in the GM-CSF free X-VIVO 15 medium only (500 μl/well). After 3 hours of incubation, macrophages were lysed with PBS + 4% Triton X-100 solution. Control wells with yeast cells only were treated similarly. Finally, the lysates and the cells were serially diluted, plated on YPD plates and incubated for 2 days at 30°C. After incubation, the number of CFUs was determined and the recovered CFUs from each strain were compared. The killing efficiency was calculated as follows: (number of live *Candida* cells in control wells – number of live *Candida* cells in co-cultures)/number of live *Candida* cells in control wells × 100. The experiments were performed with PBMC-DMs derived from five independent donors with triplicates.

### Phagocytosis assay using flow cytometry

For the phagocytosis assays, *Candida* cells were labeled with the fluorescent dye Alexa Fluor 447 carboxylic acid succimidyl ester (Invitrogen) and measured using the FlowSight Imaging flow cytometer (Amnis). For labeling, the yeast cell suspension (100 µl containing 10^9^ cells) was treated with 11 μl of Na_2_CO_3_ (1 M, pH 10) and 2 μl Alexa Fluor 447 (1 mg/ml in DMSO) was added followed by incubation for 1 hour at room temperature, carefully protected from light. After the incubation, the cells were washed four times with PBS and adjusted to the proper concentration of 2.5 × 10^6^ (MOI of 1:5). Human PBMC-DMs (5 × 10^5^/well) were infected with labeled *Candida* cells and kept for 2 hours at 37°C with 5% CO_2_. Then, the non-phagocytosed *Candida* cells were washed with PBS and the yeast containing macrophages were detached from the cell culture plates with TrypLETM Express solution (Gibco). Finally, macrophages were collected in FACS buffer (0.5% FBS in PBS), centrifuged and dissolved in 200 μl PBS and measurement using the flow cytometer. The obtained raw data was analyzed by the IDEAS Software (Amnis). The experiments were performed with PBMC-DMs derived from five independent donors.

### Phagocytosis competition assay

GFP (wild type) and mCherry (mutant strains) expressing *C. parapsilosis* strains were grown overnight in YPD at 30°C at 200 rpm. Next day, the cells were harvested by centrifugation at 3000 rpm for 5 mins and washed with PBS. PBMC-DMs were infected with a mixture of fungal cells (wild type and a mutant strain, mixed in a ratio of 1:1), and were co-incubated at 37°C for 2 hours. Then the co-culture was incubated and then washed 2–3 times with PBS before visualized under the fluorescent microscope [38]. At least 300 macrophages and 150 yeast cells were counted for each experiment and the experiments were performed with macrophages derived from three independent donors.

### LDH measurement

We determined the release of lactate dehydrogenases from the supernatant, as an indicator of host cell death, by the LDH cytotoxicity detection kit (Takara) according to the manufacturer’s instructions. Cytotoxicity was calculated after subtracting the absorbance value of the infected control from all the test samples: cytotoxicity (%) is calculated as follows: (OD_Experimental value_/OD_Positive control_) × 100. Supernatant collected from the cells treated with 1% TritonX-100 was used as positive control. The experiments were performed with PBMC-DMs generated from five independent donors.

### Analysis of phagosome lysosome fusion by FACS

For phagosome-lysosome fusion analysis by quantitative imaging (Amnis Flow Sight), the *Candida* cells were labeled with the fluorescent dye pHrodo® (Invitrogen). First, the yeast cell suspension (100 µl containing 10^9^ cells) was treated with 11 μl of Na_2_CO_3_ (1 M, pH 10) and then 2 μl pHrodo (1 mg/ml in DMSO) was added followed by incubation for 1 hr at room temperature in the dark. After the incubation, cells were washed 4 times with HBSS buffer (Hank’s Balanced Salt Solution) (LONZA) and adjusted to the proper concentration (see phagocytosis assay protocol). Human PBMC-derived macrophages were infected with labeled *Candida* cells at a 1:5 ratio and kept for 2 hrs to allow phagocytosis. Then, the non-phagocytosed *Candida* cells were washed with 1xPBS and macrophages were detached from cell culture plates by TrypLETM Express solution (Gibco). Finally, macrophages were collected in FACS buffer (0.5% FBS in 1xPBS), centrifuged and dissolved in 1xPBS. Phago-lysosome fusion was then measured by Amnis FlowSight. Data were analyzed by the IDEAS Software (Amnis).

### Cytokine measurement by ELISA

The concentration of different cytokines was analyzed by commercial ELISA kits according to the manufacturer’s instructions. The IL-1β, IL-1ra, IL-6, TNFα, IL-10 and IL-8 ELISA kits were obtained from R&D Systems (Abingdon, United Kingdom). For the pro-IL1β measurement, macrophages were lysed by repeated freeze thaw cycles and then the supernatant was collected [39]. The experiments were performed with both PBMCs and macrophages derived from peripheral blood mononuclear cells (PBMC-DMs) were used from at least five independent donors.

### *Mouse model of systemic* Candida *infection and fungal burden*

For the experiments, a non-lethal experimental mouse model of disseminated candidiasis was used [40]. Briefly, groups of five 8–12-weeks-old female Balb/c mice (22 – 27 g. of weight) were infected with 2 × 10^7^/100 μl *C. parapsilosis* cells via the lateral tail vein using a syringe with a 32-gauge needle. A group of control mice was injected with 100 µl of sterile PBS. Two independent experiments were performed. The mice were maintained with sterile water and food *ad libitum*. Three days following infection, animals were euthanized, and their livers, kidneys and spleens were aseptically removed. The organs were weighed and homogenized in sterile PBS with a tissue grinder. CFUs were determined after 48 hours of incubation at 30°C, and CFU/g tissue was calculated.

## Ethics statement

The *in vivo* mouse infection experiments were performed according to National (1998. XXVIII; 40/2013) and European (2010/63/EU) animal ethics guidelines. The experimental protocols were approved by the Animal Experimentation and Ethics Committee of the Biological Research Centre of the Hungarian Academy of Sciences and the Hungarian National Animal Experimentation and Ethics Board (clearance number: XVI./03521/2011.). The University of Szeged granted permissions XII./00455/2011 and XVI./3652/2016 to work with mice.

For PBMC isolation, blood was collected from healthy individuals. The Institutional Human Medical Biological Research Ethics Committee of the University of Szeged gave approval for the procedure and the respective consent documents. Healthy individuals provided written informed consent. The experiments were performed in accordance with guidelines and regulations of the Ethics Committee of University of Szeged and the experimental protocols were approved by the same institutional committee.

## Statistical analysis

Unpaired t-tests were used to determine differences between groups in case of phagocytosis assay, killing assay, LDH assay and phagocytic competition assay. Differences were considered statistically significant at P ≤ 0.05. One-way ANOVA with Dunnett’s Post-Hoc test was used to determine the differences between groups in case of eicosanoid analysis, cytokine analysis and *in vivo* experiment. Graph Pad Prism 6 software was used to perform the statistical analysis.
