# Supplementary material for: Eicosanoid biosynthesis influences the virulence of Candida parapsilosis
Source: Virulence. 2018 Jul 27;9(1):1019–35. doi: 10.1080/21505594.2018.1475797 (PMC6086292; doi:10.1080/21505594.2018.1475797)
Supplement: Supplemental Material [file kvir-09-01-1475797-s001.docx]

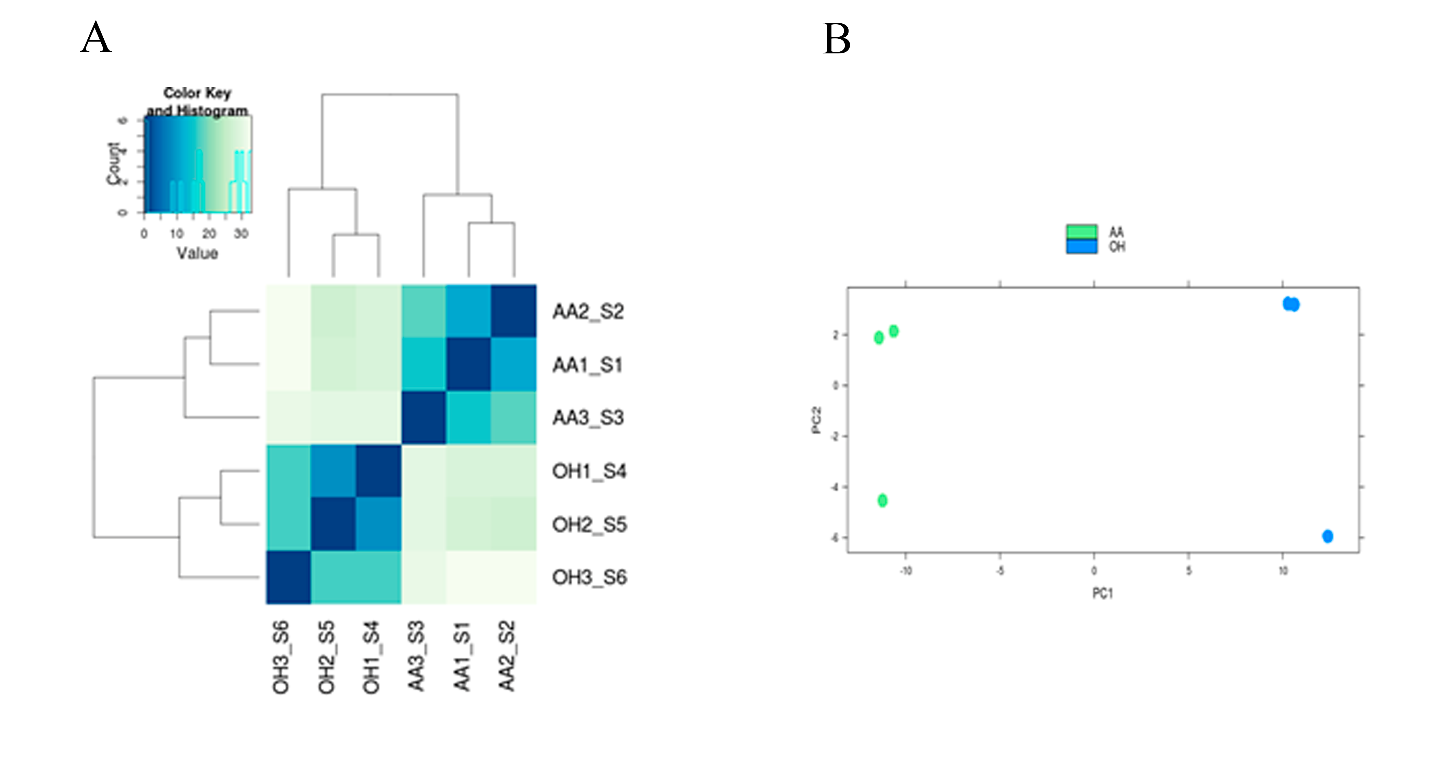


**Fig S1: Hierarchical clustering and principal component analysis of the RNA-Seq data**

Data Quality was evaluated via both hierarchical clustering (A) and Principal Component Analysis (B). Hierarchical clustering (hclust model in R) was used to illustrate the relation between the samples (S1, S2, S3, etc.) with a clear clustering of the replicates against the conditions, showing that the primary effect influencing the data was the exposure, not biological noise. Principal component analysis (prcomp model in R) was used to examine the primary variance of the samples. For both the hierarchical clustering and Principal Component Analysis, the analysis shows that the change between arachidonic acid (AA) treatment and samples without pretreatment [ethanol only, zero amount Arachidonic Acid versus background exposure increase (OH)] caused the primary variance.





**Fig S2: External eicosanoid profile of the *102550∆/∆, 205500∆/∆, 807700∆/∆* deletion mutants.**

*C. parapsilosis* CLIB 214 wild type strain and the three null mutant strains of *CPAR2_102550,* *CPAR2_205500* and *CPAR2_807700* were grown for 24hrs at 30 °C in the presence of 100 μM arachidonic acid in PBS. The eicosanoid profiles were determined as described. These did not show any significant change in the amount of PGD_2_, PGE_2_, 15-keto-PGE_2_ and 5D2-IsoP.

**Table S1: List of upregulated genes**

| **CPAR GeneID** | **Fold Change** | ***Candida albicans* homologue** | **Predicted function** |
| --- | --- | --- | --- |
| CPAR2_700300 | 4.47451615 | *C1_14020W_A* | Has domain(s) with predicted Rho GDP-dissociation inhibitor activity and cytoplasm localization |
| CPAR2_300010 | 3.79312472 | *CDR1* | Pseudogene |
| CPAR2_105530 | 3.67087524 | *DUR3* | Ortholog(s) have putrescine transmembrane transporter activity, spermidine transmembrane transporter activity, urea transmembrane transporter activity and role in putrescine transport, spermidine transport, urea transport |
| CPAR2_402870 | 3.65542755 | *MEP2* | Ortholog(s) have high-affinity secondary active ammonium transmembrane transporter activity, methylammonium transmembrane transporter activity |
| CPAR2_703250 | 3.20780930 |  | Protein of unknown function; expression increased in fluconazole and voriconazole resistant strains |
| CPAR2_805410 | 3.17580505 | *FAA2-3* | Has domain(s) with predicted catalytic activity and role in metabolic process |
| CPAR2_105520 | 3.13796806 | *DUR1,2* | Ortholog(s) have allophanate hydrolase activity, urea carboxylase activity, role in cellular response to alkaline pH, nitrogen utilization, pathogenesis, urea catabolic process and cytoplasm localization |
| CPAR2_701900 | 3.12965309 | *CR_07700W_A* | Has domain(s) with predicted protein serine/threonine kinase activity, transferase activity, transferring phosphorus-containing groups activity and role in protein phosphorylation |
| CPAR2_304370 | 3.04396652 | *CDR1* | Has domain(s) with predicted ATP binding, ATPase activity, ATPase activity, coupled to transmembrane movement of substances, nucleoside-triphosphatase activity, nucleotide binding activity and role in drug export, transport |
| CPAR2_806280 | 2.99134972 | *PHO13* | Has domain(s) with predicted hydrolase activity and role in metabolic process |
| CPAR2_808510 | 2.79452119 | *C3_06730W_A* | Ortholog(s) have glyoxysome localization |
| CPAR2_800690 | 2.72903906 | *CR_02570C_A* | Ortholog(s) have triglyceride lipase activity, role in triglyceride catabolic process and peroxisomal matrix localization |
| CPAR2_806900 | 2.71800712 | *C3_03570C_A* | Has domain(s) with predicted oxidoreductase activity and role in oxidation-reduction process |
| CPAR2_100490 | 2.71306215 | *GIT3* | Ortholog(s) have glycerophosphodiester transmembrane transporter activity and role in glycerophosphodiester transport |
| CPAR2_603800 | 2.71163940 | *CDR1* | Has domain(s) with predicted ATP binding, ATPase activity, ATPase activity, coupled to transmembrane movement of substances, nucleoside-triphosphatase activity, nucleotide binding activity and role in drug export, transport |
| CPAR2_501010 | 2.58423681 | *XUT1* | Ortholog(s) have purine nucleobase transmembrane transporter activity, role in purine nucleobase transport and fungal-type vacuole, plasma membrane localization |
| CPAR2_203620 | 2.57836162 | *C1_11530C_A* | Ortholog(s) have sulfonate dioxygenase activity and role in sulfur compound catabolic process |
| CPAR2_503690 | 2.54904104 | *FOX3* | Ortholog(s) have glyoxysome localization |
| CPAR2_800350 | 2.53962599 | *C3_04350C_A* | Has domain(s) with predicted oxidoreductase activity and role in metabolic process |
| CPAR2_807710 | 2.483301215 | *POX1-3* | Has domain(s) with predicted acyl-CoA dehydrogenase activity, acyl-CoA oxidase activity, flavin adenine dinucleotide binding, oxidoreductase activity, acting on the CH-CH group of donors activity |
| CPAR2_205500 | 2.481248275 | *ECI1* | Ortholog(s) have dodecenoyl-CoA delta-isomerase activity, role in fatty acid beta-oxidation, filamentous growth and peroxisome localization |
| CPAR2_800720 | 2.468074427 | *OPT1* | Ortholog(s) have proton-dependent oligopeptide secondary active transmembrane transporter activity, tetrapeptide transmembrane transporter activity |
| CPAR2_302780 | 2.463534476 | *C5_03690W_A* |  |
| CPAR2_103200 | 2.342975463 | *FUR4* | Pseudogene |
| CPAR2_602390 | 2.338988982 | *C6_01420C_A* | Putative oxidoreductase; expression increased in fluconazole and voriconazole resistant strains |
| CPAR2_405290 | 2.29177377 | *CDR1* | Ortholog(s) have ATP binding, drug binding, fluconazole transporter activity, phospholipid-translocating ATPase activity, xenobiotic-transporting ATPase activity |
| CPAR2_103080 | 2.287467581 | *GLX3* | Ortholog(s) have glyoxalase III activity and role in cellular response to nutrient levels, cellular response to oxidative stress, methylglyoxal catabolic process to D-lactate via S-lactoyl-glutathione |
| CPAR2_800240 | 2.284063025 | *OPT2* | Ortholog(s) have oligopeptide transmembrane transporter activity and role in nitrogen utilization, oligopeptide transmembrane transport |
| CPAR2_700870 | 2.244383043 | *PGA6* | Putative GPI-anchored cell wall protein; ortholog of C. albicans orf19.4765/PGA6; expression increased in fluconazole and voriconazole resistant strains |
| CPAR2_102550 | 2.191941927 | *FAA21* | Ortholog(s) have long-chain fatty acid-CoA ligase activity, medium-chain fatty acid-CoA ligase activity, very long-chain fatty acid-CoA ligase activity and role in long-chain fatty acid metabolic process |
| CPAR2_304270 | 2.190063355 | *GRP2* | Similar to S. cerevisiae Gre2p (methylglyoxal reductase); expression increased in fluconazole and voriconazole resistant strains |
| CPAR2_204220 | 2.165445494 | *ALK8* | Ortholog(s) have alkane 1-monooxygenase activity, oxygen binding activity and role in fatty acid omega-oxidation, lauric acid metabolic process |
| CPAR2_800020 | 2.107629768 | *POT1* | Ortholog(s) have acetyl-CoA C-acyltransferase activity, mRNA binding activity, role in fatty acid beta-oxidation and mitochondrial intermembrane space, peroxisomal matrix localization |
| CPAR2_209750 | 2.094149889 | *C2_05640W_A* | Ortholog(s) have role in filamentous growth |
| CPAR2_209850 | 2.07919037 | *TES15* | Ortholog(s) have glyoxysome localization |
| CPAR2_503040 | 1.999850027 | *FOX2* | Ortholog(s) have 3-hydroxyacyl-CoA dehydrogenase activity, 3-hydroxybutyryl-CoA epimerase activity, enoyl-CoA hydratase activity |
| CPAR2_106260 | 1.978403933 | *C1_02270C_A* | Ortholog(s) have cytosol, nucleus localization |
| CPAR2_109800 | 1.961847171 | *C1_01140C_A* |  |
| CPAR2_208430 | 1.938195717 | *COI1* | Ortholog(s) have cell surface, extracellular region localization |
| CPAR2_405280 | 1.917931809 | *CDR1* | Ortholog(s) have role in fluconazole transport |
| CPAR2_213670 | 1.890075591 | *PGA52* | Ortholog(s) have anchored component of plasma membrane, endoplasmic reticulum, fungal-type cell wall, mitochondrion localization |
| CPAR2_304110 | 1.846691183 | *C5_00390C_A* |  |
| CPAR2_801040 | 1.821295786 | *CR_02570C_A* | Ortholog(s) have triglyceride lipase activity, role in triglyceride catabolic process and peroxisomal matrix localization |
| CPAR2_207300 | 1.814253043 | *C1_10360C_A* | Protein of unknown function; expression increased in fluconazole and voriconazole resistant strains |
| CPAR2_807700 | 1.806876842 | *POX1-3* | Ortholog(s) have role in fatty acid beta-oxidation and peroxisomal matrix localization |
| CPAR2_107140 | 1.800815294 | *C1_04150C_A* | Has domain(s) with predicted hydrolase activity |
| CPAR2_206630 | 1.795626243 | *C2_05130W_A* | Protein of unknown function; expression increased in fluconazole and voriconazole resistant strains |
| CPAR2_503540 | 1.780401329 | *PTR22* | Ortholog(s) have dipeptide transmembrane transporter activity, tripeptide transporter activity and role in dipeptide transmembrane transport, tripeptide transport |
| CPAR2_208960 | 1.767915495 | *C1_06920C_A* |  |
| CPAR2_600460 | 1.742597811 | *HGT10* | Has domain(s) with predicted substrate-specific transmembrane transporter activity, transmembrane transporter activity, role in transmembrane transport and integral component of membrane, membrane localization |
| CPAR2_802230 | 1.739837554 | *CR_03540W_A* | Ortholog(s) have cytoplasm, lipid particle localization |
| CPAR2_700290 | 1.695825557 | *C1_14020W_A* | Has domain(s) with predicted Rho GDP-dissociation inhibitor activity and cytoplasm localization |
| CPAR2_204330 | 1.660923151 | *HSP104* | Ortholog(s) have ADP binding, ATP binding, ATPase activity, coupled, chaperone binding, misfolded protein binding, unfolded protein binding activity |
| CPAR2_303510 | 1.646877415 | *TAC1* | Ortholog(s) have sequence-specific DNA binding, transcription coactivator activity, transcription factor activity, RNA polymerase II distal enhancer sequence-specific binding activity |
| CPAR2_603600 | 1.639560294 | *FET31* | Ortholog(s) have role in cellular response to drug, ferrous iron import across plasma membrane and endoplasmic reticulum, plasma membrane localization, prostaglandin biosynthesis |
| CPAR2_500590 | 1.629695081 | *GAT1* | Ortholog(s) have sequence-specific DNA binding, transcription factor activity, RNA polymerase II transcription factor binding and transcriptional activator activity |
| CPAR2_208020 | 1.628387873 | *C1_07160C_A* |  |
| CPAR2_600930 | 1.614743718 | *C6_03370W_A* |  |
| CPAR2_208190 | 1.606410269 | *PHO84* | Ortholog(s) have phosphate:proton symporter activity |
| CPAR2_208160 | 1.605557038 | *PHO84* | Ortholog(s) have inorganic phosphate transmembrane transporter activity, manganese ion transmembrane transporter activity, phosphate:proton symporter activity, selenite:proton symporter activity |
| CPAR2_406710 | 1.595465854 | *ARE2* | Ortholog(s) have ergosterol O-acyltransferase activity, role in ergosterol metabolic process and endoplasmic reticulum localization |
| CPAR2_805510 | 1.593655443 | *LEU2* | Ortholog(s) have 3-isopropylmalate dehydrogenase activity, role in glyoxylate cycle, leucine biosynthetic process, pathogenesis and biofilm matrix, cytosol localization |
| CPAR2_404310 | 1.592215777 | *MDH1-3* | Ortholog(s) have L-malate dehydrogenase activity, mRNA binding activity, role in NADH regeneration, fatty acid beta-oxidation and peroxisomal importomer complex, peroxisomal matrix localization |
| CPAR2_105480 | 1.569347115 | *C1_04460C_A* | Protein of unknown function; expression increased in fluconazole and voriconazole resistant strains |
| CPAR2_303750 | 1.535070361 | *HMS1* | Ortholog(s) have transcription regulatory region DNA binding activity |
| CPAR2_700030 | 1.530620174 | *CDR1* | Has domain(s) with predicted ATP binding, ATPase activity, ATPase activity, coupled to transmembrane movement of substances, nucleoside-triphosphatase activity, nucleotide binding activity and role in drug export, transport |
| CPAR2_807590 | 1.530311577 | *C3_04730C_A* |  |
| CPAR2_301610 | 1.510390828 | *C7_00350C_A* |  |

**Table S2: List of strains used in the study**

| **Strain Name** | **Parent** | **CPAR GeneID** | **Genotype** | **Reference** |
| --- | --- | --- | --- | --- |
| GA1 |  |  |  | Type Strain |
| CLIB214 |  |  |  | Type Strain |
| CPL2H1 |  |  | *leu2::FRT/leu2::FRT, his1::FRT/his1::FRT* | Holland et al,2014 |
| *102550Δ/Δ* | CPL2H1 | CPAR2_102550 | *leu2::FRT/leu2::FRT, his1::FRT/his1::FRT, cpar2_102550::LEU2/cpar2_102550::HIS1* | This study |
| *205500Δ/Δ* | CPL2H1 | CPAR2_205500 | *leu2::FRT/leu2::FRT, his1::FRT/his1::FRT, cpar2_205500::LEU2/cpar2_205500::HIS1* | This study |
| *603600Δ/Δ* | CPL2H1 | CPAR2_603600 | *leu2::FRT/leu2::FRT, his1::FRT/his1::FRT, cpar2_603600::LEU2/cpar2_603600::HIS1* | This study |
| *800020Δ/Δ* | CPL2H1 | CPAR2_800020 | *leu2::FRT/leu2::FRT, his1::FRT/his1::FRT, cpar2_800020::LEU2/cpar2_800020::HIS1* | This study |
| *807700Δ/Δ* | CPL2H1 | CPAR2_807700 | *leu2::FRT/leu2::FRT, his1::FRT/his1::FRT, cpar2_807700::LEU2/cpar2_807700::HIS1* | This study |
| *807710Δ/Δ* | CPL2H1 | CPAR2_807710 | *leu2::FRT/leu2::FRT, his1::FRT/his1::FRT, cpar2_807710::LEU2/cpar2_807710::HIS1* | This study |
| CPL2H1-GFP | CPL2H1 |  | *leu2::FRT/leu2::FRT, his1::FRT/his1::FRT, CpNEUT5L/Cpneut5l : : GFP-LEU2* | This study |
| *603600Δ/*Δ*-mCherry* |  |  | *leu2::FRT/leu2::FRT, his1::FRT/his1::FRT, cpar2_603600::LEU2/cpar2_603600::HIS1,CpNEUT5L/Cpneut5l : : mCHERRY-NAT1* | This study |
| *800020Δ/Δ-mCherry* |  |  | *leu2::FRT/leu2::FRT, his1::FRT/his1::FRT, cpar2_800020::LEU2/cpar2_800020::HIS1,CpNEUT5L/Cpneut5l : : mCHERRY-NAT1* | This study |
| *807710Δ/Δ-mCherry* |  |  | *leu2::FRT/leu2::FRT, his1::FRT/his1::FRT, cpar2_807710::LEU2/cpar2_807710::HIS1,CpNEUT5L/Cpneut5l : : mCHERRY-NAT1* | This study |

**Table S3: List of primers used in the study**

| **Primer Name** | **Sequence (5' to 3')** |
| --- | --- |
| 102550 RTF | ACAGGTGCAAATCCAAAAGG |
| 102550 RTR | CGGCATCTTGCTTTGGTAAT |
| 205500 RTF | TTGGAAGAGACCCAGTCGTT |
| 205500 RTR | ACTGGCCAATTCAGCAAATC |
| 603600 RTF | AGTAGGTGTGCCATGGGAAG |
| 603600 RTR | GTGAATCCAGTGGGCAATCT |
| 800020 RTF | AACGTTCATTTGCCGAAAAG |
| 800020 RTR | GCTGGACCAACACCCATAAT |
| 807700 RTF | TTGATTGTCGATGGCAAAGA |
| 807700 RTR | CAATGTCACCAACGGAAACA |
| 807710 RTF | ACGGTACTGCCGATCAAATC |
| 807710 RTR | ATCCGTGAGCCAACTCAGTC |
| qTub4F | gaacacttatgccgaggacaac |
| qTub4R | ACTCTCACCACTGACTCCTTGC |
| 102550 Pri1 | GTCATCGATATTTGCCATCCTAG |
| 102550 Pri3 | cacggcgcgcctagcagcggGGTGCCATGATAACCGGAG |
| 102550 Pri5 | gcagggatgcggccgctgacAGTGCTACTCTGACCTACTTagctcggatccactagtaacg |
| 102550 Pri4 | gtcagcggccgcatccctgcGTTGATGTTGAGCCCTTATCGG |
| 102550 Pri6 | CGAAGCGATTCGAACATCTTC |
| 205500 Pri1 | CCGAAGGTGAAGCAATCGTTAC |
| 205500 Pri3 | cacggcgcgcctagcagcggGGCGCATTTACCTTTAACTTCG |
| 205500 Pri5 | gcagggatgcggccgctgacAGGCACTTGCTCCAGGACTTagctcggatccactagtaacg |
| 205500 Pri4 | gtcagcggccgcatccctgcGCCAAAGAGGCGTAACTAGA |
| 205500 Pri6 | GGTTAAGGCTCATATGGCGG |
| 603600 Pri1 | GCTTTCCAATGTCGTAGTTTACC |
| 603600 Pri3 | cacggcgcgcctagcagcggCGAGTGCCAATATAGTGAGG |
| 603600 Pri5 | gcagggatgcggccgctgacACGAGGTCTTGCATCTACTTagctcggatccactagtaacg |
| 603600 Pri4 | gtcagcggccgcatccctgcGCCAGAGACTTGGATGTTG |
| 603600 Pri6 | GCGAAATAGCACTCCCATTATC |
| 800020 Pri1 | CGTTGGTAGCTCGGATGTAA |
| 800020 Pri3 | cacggcgcgcctagcagcggGTTGTTGAGTGATTCCATGGTG |
| 800020 Pri5 | gcagggatgcggccgctgacATGGCCGTGTCAGCCTACTTagctcggatccactagtaacg |
| 800020 Pri4 | gtcagcggccgcatccctgcCGGTGGTGCTATTGCATTAGG |
| 800020 Pri6 | CAAATCCTCGATGGTGGGTGTC |
| 807700 Pri1 | AGCGGTATACTCCACCTGG |
| 807700 Pri3 | cacggcgcgcctagcagcggGGCCCTTGCTTAAACTGACAG |
| 807700 Pri5 | gcagggatgcggccgctgacACGCGCTGCTTAGATTACTTagctcggatccactagtaacg |
| 807700 Pri4 | gtcagcggccgcatccctgcGCTATT GGTAAATACGACGGTG |
| 807700 Pri6 | CGTGTGTTTGTGTTGCTCTAT |
| 807710 Pri1 | AGAATTGGCGATCGAAAACAACG |
| 807710 Pri3 | cacggcgcgcctagcagcggGCTGTGAATGGAACCGACAT |
| 807710 Pri5 | gcagggatgcggccgctgacAAGCTGACCTTCTTGCACTTagctcggatccactagtaacg |
| 807710 Pri4 | gtcagcggccgcatccctgcGTCCAAGTAAACCCGACTTCTTG |
| 807710 Pri6 | GGGGGTACTTGTTATCAGGTGA |
| Univ_Primer_2 | ccgctgctaggcgcgccgtgACCAGTGTGATGGATATCTGC |
| His Chk1 | AAAATCAATGGGCATTCTCG |
| His Chk2 | TGGGAAGCAGACATTCAACA |
| Leu2 Chk1 | GAAGTTGGTGACGCGATTGT |
| Leu2 Chk2 | TTCCCCTTCAATGTATGCAA |

**Table S4: MRM characteristics of the monitored eicosanoids**

| **Name entry** | **Compound** | **Lipid Maps**  **ID** | **Retention time (min)** | **m/z in Q1** | **m/z in Q3** | **Declustering potential [V]** | **Collision energy [V]** | **Collision cell exit potential [V]** | **Group** | **_2 method** | **_5 method** |
| --- | --- | --- | --- | --- | --- | --- | --- | --- | --- | --- | --- |
| ALA_2 | ALA | LMFA01030152 | 8.6 | 277 | 182.1 | -55 | -24 | -7 | Polyunsaturated fatty acids | 0 | 1 |
| GLA | GLA | LMFA01030141 | 8.6 | 277 | 179.1 | -75 | -20 | -11 | Polyunsaturated fatty acids | 0 | 1 |
|  | 13,14-dihydro-15-keto-PGE2 | LMFA03010031 | 4.8 | 351.1 | 235 | -45 | -30 | -13 | Prostaglandins (PG) | 1 | 1 |
| 19(20)EpDPA | 19(20)-EpDPA | LMFA04000038 | 8.1 | 343.1 | 281.1 | -70 | -16 | -11 | Epoxydocosapentaenoic acids | 0 | 1 |
|  | 11(12)-EET | LMFA03080004 | 8.2 | 318.9 | 166.9 | -90 | -18 | -19 | Epoxyeicosatrienoic acids (EET) | 1 | 1 |
|  | 14(15)-EET | LMFA03080005 | 8.1 | 319 | 218.9 | -5 | -16 | -55 | Epoxyeicosatrienoic acids (EET) | 1 | 1 |
|  | 8(9)-EET | LMFA03080003 | 8.2 | 319 | 154.9 | -60 | -18 | -13 | Epoxyeicosatrienoic acids (EET) | 1 | 1 |
|  | 10-HDHA | LMFA04000027 | 7.9 | 343.1 | 153 | -25 | -20 | -15 | Hydroxydocosahexaenoic acids (HDHA) | 1 | 1 |
| 14(S)-HDHA | 14(S)-HDHA | LMFA04000058 | 8.0 | 343.1 | 204.9 | -60 | -18 | -27 | Hydroxydocosahexaenoic acids (HDHA) | 0 | 1 |
|  | 17-HDHA | LMFA04000072 | 7.9 | 343.1 | 245 | -65 | -16 | -15 | Hydroxydocosahexaenoic acids (HDHA) | 1 | 1 |
| 4-HDHA | 4-HDHA | LMFA04000058 | 8.2 | 343.1 | 101 | -50 | -18 | -9 | Hydroxydocosahexaenoic acids (HDHA) | 0 | 1 |
|  | 7-HDHA | LMFA04000025 | 8.0 | 343.1 | 141.1 | -85 | -18 | -23 | Hydroxydocosahexaenoic acids (HDHA) | 1 | 1 |
|  | 19,20-DiHDPA | LMFA04000043 | 7.4 | 361.1 | 273 | -55 | -22 | -15 | Hydroxydocosapentaenoic acids (HDPA) | 1 | 1 |
|  | 7,17-DiHDPA | N/A | 7.0 | 361.1 | 198.9 | -45 | -26 | -23 | Hydroxydocosapentaenoic acids (HDPA) | 1 | 1 |
| 12-HEPE | 12-HEPE | LMFA03070031 | 7.6 | 317 | 179 | -60 | -18 | -17 | Hydroxyeicosapentaenoic acids (HEPE) | 0 | 1 |
|  | 15-HEPE | LMFA03070009 | 7.5 | 317.1 | 219 | -65 | -18 | -19 | Hydroxyeicosapentaenoic acids (HEPE) | 1 | 1 |
|  | 18-HEPE | LMFA03070038 | 7.4 | 317.1 | 259 | -5 | -16 | -7 | Hydroxyeicosapentaenoic acids (HEPE) | 1 | 1 |
| 5-HEPE | 5-HEPE | LMFA03070027 | 7.7 | 317 | 114.9 | -55 | -18 | -11 | Hydroxyeicosapentaenoic acids (HEPE) | 0 | 1 |
|  | 11-HETE | LMFA03060003 | 7.9 | 319.1 | 167 | -70 | -22 | -15 | Hydroxyeicosatetraenoic acids (HETE) | 1 | 1 |
|  | 12-HETE | LMFA03060007 | 7.9 | 319.1 | 179 | -65 | -20 | -23 | Hydroxyeicosatetraenoic acids (HETE) | 1 | 1 |
|  | 14,15-diHETE | LMFA03060077 | 7.0 | 335.1 | 207 | -65 | -24 | -21 | Hydroxyeicosatetraenoic acids (HETE) | 1 | 1 |
|  | 15-HETE | LMFA03060001 | 7.8 | 319.1 | 219.1 | -55 | -18 | -9 | Hydroxyeicosatetraenoic acids (HETE) | 1 | 1 |
|  | 17-OH-DH-HETE | N/A | 8.2 | 347.1 | 247 | -110 | -22 | -27 | Hydroxyeicosatetraenoic acids (HETE) | 1 | 1 |
| 20-HETE_2 | 20-HETE | LMFA03060009 | 7.7 | 319 | 289.1 | -70 | -24 | -15 | Hydroxyeicosatetraenoic acids (HETE) | 0 | 1 |
|  | 5,15-diHETE | LMFA03060010 | 6.8 | 335 | 173.1 | -55 | -20 | -11 | Hydroxyeicosatetraenoic acids (HETE) | 1 | 1 |
|  | 5-HETE | LMFA03060002 | 8.0 | 319.1 | 115 | -65 | -18 | -11 | Hydroxyeicosatetraenoic acids (HETE) | 1 | 1 |
|  | 8(S),15(S)-diHETE | LMFA03060050 | 6.7 | 335.1 | 207.9 | -55 | -22 | -17 | Hydroxyeicosatetraenoic acids (HETE) | 1 | 1 |
|  | 8-HETE | LMFA03060006 | 7.9 | 319.1 | 154.9 | -70 | -20 | -19 | Hydroxyeicosatetraenoic acids (HETE) | 1 | 1 |
|  | 13-HoDE | LMFA02000228 | 7.7 | 295 | 194.9 | -110 | -24 | -21 | Hydroxyoctadecadienoic acids (HoDE) | 1 | 1 |
|  | 9-HoDE | LMFA02000188 | 7.7 | 295 | 171 | -130 | -22 | -7 | Hydroxyoctadecadienoic acids (HoDE) | 1 | 1 |
|  | 13-HoTrE | LMFA02000051 | 7.4 | 293 | 195 | -45 | -24 | -19 | Hydroxyoctadecatrienoic acids (HoTrE) | 1 | 1 |
|  | 9-HoTrE | LMFA02000024 | 7.4 | 293 | 170.9 | -75 | -20 | -15 | Hydroxyoctadecatrienoic acids (HoTrE) | 1 | 1 |
|  | 15-HETE-d8 | LMFA03060080 | 7.8 | 327.2 | 226 | -85 | -18 | -11 | Internal standards | 1 | 1 |
|  | DHA-d5 | LMFA01030762 | 8.8 | 332 | 288.1 | -75 | -16 | -13 | Internal standards | 1 | 1 |
|  | LTB4-d4 | LMFA03020030 | 6.9 | 339.1 | 196.9 | -70 | -22 | -19 | Internal standards | 1 | 1 |
|  | PGE2-d4 | LMFA03010008 | 4.9 | 355.1 | 193 | -50 | -26 | -17 | Internal standards | 1 | 1 |
|  | 17-F2t-dihomo-IsoP | LMFA03110167 | 6.1 | 381.1 | 318.9 | -115 | -32 | -41 | Isoprostanes (IsoP) and neuroprostanes (NeuroP) | 1 | 1 |
|  | 4-F4t-NeuroP | N/A | 5.2 | 377.1 | 270.9 | -15 | -26 | -13 | Isoprostanes (IsoP) and neuroprostanes (NeuroP) | 1 | 1 |
|  | 5-F3t-IsoP | N/A | 3.8 | 351 | 114.8 | -95 | -26 | -13 | Isoprostanes (IsoP) and neuroprostanes (NeuroP) | 1 | 1 |
| 12-KETE | 12-KETE | LMFA03060019 | 7.9 | 317 | 153 | -60 | -22 | -9 | Keto-eicosatetraenoic acids (KETE/OxoETE) | 0 | 1 |
| 15-KETE | 15-KETE | LMFA03060051 | 7.8 | 317 | 113 | -10 | -22 | -5 | Keto-eicosatetraenoic acids (KETE/OxoETE) | 0 | 1 |
| 5-KETE | 5-KETE | LMFA03060011 | 8.1 | 317 | 203.1 | -70 | -24 | -11 | Keto-eicosatetraenoic acids (KETE/OxoETE) | 0 | 1 |
|  | 20-OH-LTB4 | LMFA03020018 | 4.0 | 351.1 | 195 | -60 | -24 | -17 | Leukotrienes (LT) | 1 | 1 |
|  | 6-trans-12-epi-LTB4 | LMFA03020014 | 6.8 | 335.1 | 194.9 | -80 | -22 | -25 | Leukotrienes (LT) | 1 | 1 |
|  | 6-trans-LTB4 | LMFA03020013 | 6.7 | 335.1 | 194.9 | -105 | -22 | -11 | Leukotrienes (LT) | 1 | 1 |
|  | LTB4 | LMFA03020001 | 6.9 | 335.1 | 195 | -65 | -22 | -21 | Leukotrienes (LT) | 1 | 1 |
|  | LTD4 | LMFA03020006 | 6.7 | 495.1 | 177 | -70 | -28 | -19 | Leukotrienes (LT) | 1 | 1 |
|  | LTE4 | LMFA03020002 | 7.0 | 438.1 | 333.1 | -55 | -26 | -15 | Leukotrienes (LT) | 1 | 1 |
|  | AT-LXA4 | LMFA03040003 | 5.6 | 351.1 | 114.9 | -20 | -22 | -11 | Lipoxins (LX) | 1 | 1 |
|  | LXA4 | LMFA03040001 | 5.5 | 351.1 | 114.8 | -40 | -20 | -11 | Lipoxins (LX) | 1 | 1 |
|  | LXB4 | LMFA03040002 | 5.1 | 351.1 | 220.9 | -60 | -22 | -13 | Lipoxins (LX) | 1 | 1 |
|  | Mar1 | LMFA04050001 | 7.0 | 359.2 | 250.2 | -65 | -20 | -13 | Maresins (MaR) | 1 | 1 |
|  | 7(S)-MaR1 | N/A | 6.6 | 359.1 | 249.9 | -20 | -20 | -19 | Maresins (MaR) | 1 | 1 |
|  | AA | LMFA01030001 | 8.8 | 303 | 205.1 | -155 | -20 | -11 | Polyunsaturated fatty acids | 1 | 1 |
|  | AdA | LMFA01030178 | 9.1 | 331.1 | 233 | -130 | -22 | -11 | Polyunsaturated fatty acids | 1 | 1 |
|  | ALA (+ GLA) | LMFA01030152 (LMFA01030141) | 8.6 | 277 | 233 | -90 | -22 | -29 | Polyunsaturated fatty acids | 1 | 1 |
| DGLA | DGLA | LMFA01030158 | 9.0 | 305.1 | 261.2 | -85 | -22 | -13 | Polyunsaturated fatty acids | 0 | 1 |
|  | DHA | LMFA01030185 | 8.8 | 327.1 | 229.2 | -115 | -18 | -11 | Polyunsaturated fatty acids | 1 | 1 |
|  | DPAn-3 | LMFA04000044 | 8.9 | 329.1 | 231.1 | -50 | -20 | -17 | Polyunsaturated fatty acids | 1 | 1 |
| DPAn-6 | DPAn-6 | LMFA01030182 |  |  |  |  |  |  | Polyunsaturated fatty acids | 0 | 1 |
|  | EPA | LMFA01030759 | 8.6 | 301 | 202.9 | -125 | -18 | -21 | Polyunsaturated fatty acids | 1 | 1 |
|  | LA | LMFA01030120 | 8.8 | 279 | 261 | -115 | -28 | -13 | Polyunsaturated fatty acids | 1 | 1 |
|  | 13,14-dihydro-15-keto-PGF2alpha | LMFA03010027 | 5.4 | 353.1 | 195 | -110 | -32 | -11 | Prostaglandins (PG) | 1 | 1 |
|  | 15-deoxy-PGJ2 | LMFA03010021 | 7.3 | 315 | 203 | -50 | -28 | -19 | Prostaglandins (PG) | 1 | 1 |
|  | 15-keto-PGE2 | LMFA03010030 | 4.5 | 349 | 234.9 | -65 | -20 | -13 | Prostaglandins (PG) | 1 | 1 |
|  | 8-iso-PGE2 | LMFA03110003 | 4.6 | 351.1 | 271 | -5 | -24 | -19 | Prostaglandins (PG) | 1 | 1 |
|  | 8-iso-PGF2alpha | LMFA03110001 | 4.5 | 353.1 | 193 | -135 | -34 | -11 | Prostaglandins (PG) | 1 | 1 |
|  | PGD2 | LMFA03010004 | 5.0 | 351.1 | 233 | -30 | -16 | -13 | Prostaglandins (PG) | 1 | 1 |
|  | PGE2 | LMFA03010003 | 4.9 | 351.2 | 271.1 | -50 | -22 | -21 | Prostaglandins (PG) | 1 | 1 |
|  | PGF2alpha | LMFA03010002 | 5.2 | 353.1 | 193 | -80 | -34 | -11 | Prostaglandins (PG) | 1 | 1 |
|  | PGJ2 | LMFA03010019 | 6.1 | 333 | 271 | -30 | -22 | -17 | Prostaglandins (PG) | 1 | 1 |
|  | PD1 | LMFA04040001 | 6.9 | 359.1 | 153 | -70 | -22 | -9 | Protectins (PD) | 1 | 1 |
|  | PDX | LMFA04040003 | 6.8 | 359.1 | 153 | -70 | -22 | -9 | Protectins (PD) | 1 | 1 |
|  | 18R-RvE3 | LMFA03140006 | 7.1 | 333.1 | 245 | -55 | -18 | -23 | Resolvins (Rv) | 1 | 1 |
|  | 18S-RvE3 | LMFA03140007 | 6.7 | 333.1 | 245.2 | -25 | -16 | -17 | Resolvins (Rv) | 1 | 1 |
|  | AT-RvD1 | LMFA04030005 | 5.7 | 375 | 215 | -50 | -26 | -11 | Resolvins (Rv) | 1 | 1 |
|  | RvD1 | LMFA04030011 | 5.6 | 375.1 | 215 | -50 | -26 | -11 | Resolvins (Rv) | 1 | 1 |
|  | RvD2 | LMFA04030001 | 5.3 | 375.1 | 277.1 | -60 | -18 | -15 | Resolvins (Rv) | 1 | 1 |
|  | RvE1 | LMFA03140003 | 3.8 | 349.1 | 195 | -95 | -22 | -13 | Resolvins (Rv) | 1 | 1 |
|  | RvE2 | LMFA03140011 | 6.1 | 333.1 | 114.9 | -35 | -18 | -15 | Resolvins (Rv) | 1 | 1 |
|  | TXB2 | LMFA03030002 | 4.6 | 369.1 | 169 | -55 | -24 | -15 | Thromboxanes (Tx) | 1 | 1 |
